# VAPB/ALS8 MSP Ligands Regulate Striated Muscle Energy Metabolism Critical for Adult Survival in *Caenorhabditis elegans*

**DOI:** 10.1371/journal.pgen.1003738

**Published:** 2013-09-05

**Authors:** Sung Min Han, Hajer El Oussini, Jelena Scekic-Zahirovic, Jack Vibbert, Pauline Cottee, Jeevan K. Prasain, Hugo J. Bellen, Luc Dupuis, Michael A. Miller

**Affiliations:** 1 Department of Cell, Developmental, and Integrative Biology, University of Alabama School of Medicine, Birmingham, Alabama, United States of America; 2 INSERM, U1118, Mécanismes centraux et périphériques de la neurodégénérescence, Strasbourg, France; 3 Faculté de Médecine, Fédération de Médecine Translationnelle de Strasbourg, Université de Strasbourg, UMRS1118, Strasbourg, France; 4 Department of Pharmacology and Toxicology, University of Alabama School of Medicine, Birmingham, Alabama, United States of America; 5 Howard Hughes Medical Institute, Chevy Chase, Maryland, United States of America; 6 Department of Molecular and Human Genetics, Baylor College of Medicine, Houston, Texas, United States of America; 7 Program in Developmental Biology, Baylor College of Medicine, Houston, Texas, United States of America; University of California San Francisco, United States of America

## Abstract

Mutations in VAPB/ALS8 are associated with amyotrophic lateral sclerosis (ALS) and spinal muscular atrophy (SMA), two motor neuron diseases that often include alterations in energy metabolism. We have shown that *C. elegans* and Drosophila neurons secrete a cleavage product of VAPB, the N-terminal major sperm protein domain (vMSP). Secreted vMSPs signal through Roundabout and Lar-like receptors expressed on striated muscle. The muscle signaling pathway localizes mitochondria to myofilaments, alters their fission/fusion balance, and promotes energy production. Here, we show that neuronal loss of the *C. elegans* VAPB homolog triggers metabolic alterations that appear to compensate for muscle mitochondrial dysfunction. When vMSP levels drop, cytoskeletal or mitochondrial abnormalities in muscle induce elevated DAF-16, the Forkhead Box O (FoxO) homolog, transcription factor activity. DAF-16 promotes muscle triacylglycerol accumulation, increases ATP levels in adults, and extends lifespan, despite reduced muscle mitochondria electron transport chain activity. Finally, *Vapb* knock-out mice exhibit abnormal muscular triacylglycerol levels and FoxO target gene transcriptional responses to fasting and refeeding. Our data indicate that impaired vMSP signaling to striated muscle alters FoxO activity, which affects energy metabolism. Abnormalities in energy metabolism of ALS patients may thus constitute a compensatory mechanism counterbalancing skeletal muscle mitochondrial dysfunction.

## Author Summary

ALS patients often present with systemic alterations in energy metabolism, such as dyslipidemia and hypermetabolism of unknown origin. Reduction of *Vapb* function is thought to cause motor neuron disease in *ALS8* patients and may predispose individuals to ALS, in general. We have shown that neurons secrete the N-terminal VAPB vMSP into the extracellular environment. The secreted vMSPs signal through Roundabout and Lar-like receptors on striated muscles. This neuron to muscle signaling pathway localizes mitochondria to myofilament I-bands and promotes mitochondrial function. Here we show that loss of VAPB in *C. elegans* neurons causes metabolic changes in muscles, including altered fat metabolism and elevated DAF-16 FoxO transcription factor activity. DAF-16 promotes muscle triacylglycerol accumulation, increases ATP levels, and prolongs survival in *Vapb* mutants. However, it does not influence muscle mitochondrial localization nor does it affect oxygen consumption. We also show that *Vapb* knockout mice exhibit disrupted muscular triacylglycerol and FoxO target gene transcriptional responses to fasting and refeeding. These data indicate that impaired vMSP signaling to muscle triggers an energy deficiency, which induces a protective metabolic response involving FoxO. Hence, some energy metabolism alterations observed in ALS patients might be a consequence of striated muscle mitochondrial dysfunction.

## Introduction

ALS is a lethal neurodegenerative disease characterized by the combined degeneration of lower and upper motor neurons [1]. Most ALS cases occur sporadically, but about 10% are familial. These genetic cases are caused by mutations in multiple genes, including in the *Vapb* (VAMP/synaptobrevin-associated protein B) gene. Mutations in *Vapb* lead to ALS8 that manifests as ALS or late-onset SMA, a motor neuron disease restricted to lower motor neurons [2]–[4]. While *Vapb* mutations are rare, reduced VAPB mRNA or protein levels have been reported in sporadic ALS patients, a mSOD1 ALS mouse model, and *ALS8* patient motor neurons derived from induced pluripotent stem cells [5]–[7]. Hence, a loss of VAPB might be relevant in non-*ALS8* patients.

VAPB, and its paralog VAPA, are broadly expressed type II membrane proteins that are evolutionarily conserved. These VAPs have been implicated in regulating lipid transport and homeostasis at intracellular organelle contact sites, endoplasmic reticulum (ER) dynamics, and membrane trafficking [8]–[12]. In addition to these cell autonomous functions, the VAP vMSP is cleaved from the transmembrane domain in the cytoplasm and secreted in a cell-type specific fashion [13]–[15]. Secreted vMSPs antagonize Eph receptor signaling through a direct interaction with the extracellular domain [13]. More recently, we have shown in *C. elegans* and Drosophila that neurons secrete vMSPs to regulate mitochondrial localization and function in striated muscle [15]. vMSPs interact with muscle SAX-3 Roundabout and CLR-1 Lar-like protein-tyrosine phosphatase receptors to down-regulate CLR-1 signaling. VAP loss causes uncontrolled CLR-1 Lar-like receptor activation in body wall muscle. CLR-1 stimulates actin filament assembly in the muscle belly that requires the actin-related protein 2/3 (Arp2/3) complex. These ectopic actin filaments displace mitochondria from I-bands, cause aberrant fission and fusion balance, and impair respiratory chain activity. Hence, vMSPs secreted by neurons promote muscle mitochondrial localization and function, perhaps in an effort to modulate energy homeostasis.

vMSP signaling to muscle mitochondria might be relevant for the energy balance in *ALS8* disease. Out of five *ALS8* patients studied, five had increased cholesterol levels, four had reduced HDL, three had elevated triacylglycerol levels, and one was diabetic [16]. More generally, ALS is associated with a spectrum of abnormalities in energy metabolism, including mitochondrial defects in neurons and skeletal muscle, insulin resistance, dyslipidemia, and hypermetabolism [17]. These metabolic abnormalities are positively correlated with survival. For instance, increased prediagnostic body fat is associated with decreased risk of ALS mortality [18] and in some patient populations, higher LDL/HDL ratios correlate with increased survival time [19],[20]. However, the cause(s) of the metabolic defects and their relationship to each other are not well understood.

Here we show in *C. elegans* that loss of the VAP homolog VPR-1 causes triacylglycerol (TAG) accumulation in striated body wall muscle. Mosaic analysis and tissue-specific expression studies provide compelling evidence that VPR-1 acts in neurons, not muscles to regulate fat levels. Multiple lines of evidence support the model that impaired vMSP signaling from neurons to muscle increases TAG levels in muscle. We propose that this fat metabolism alteration is part of a compensatory response mediated by the DAF-16/FoxO transcription factor. FoxO promotes muscle fat accumulation, maintains ATP levels during aging, and extends lifespan without influencing muscle mitochondrial morphology, localization, or function. Finally, we provide evidence that skeletal muscle metabolism is abnormal in *Vapb* mutant mice. Our results support the model that disrupting vMSP signaling to muscle triggers a compensatory response involving FoxO transcription factors.

## Results

### *vpr-1/vap* loss increases fat levels in adult body wall muscle

**Figure 1:**
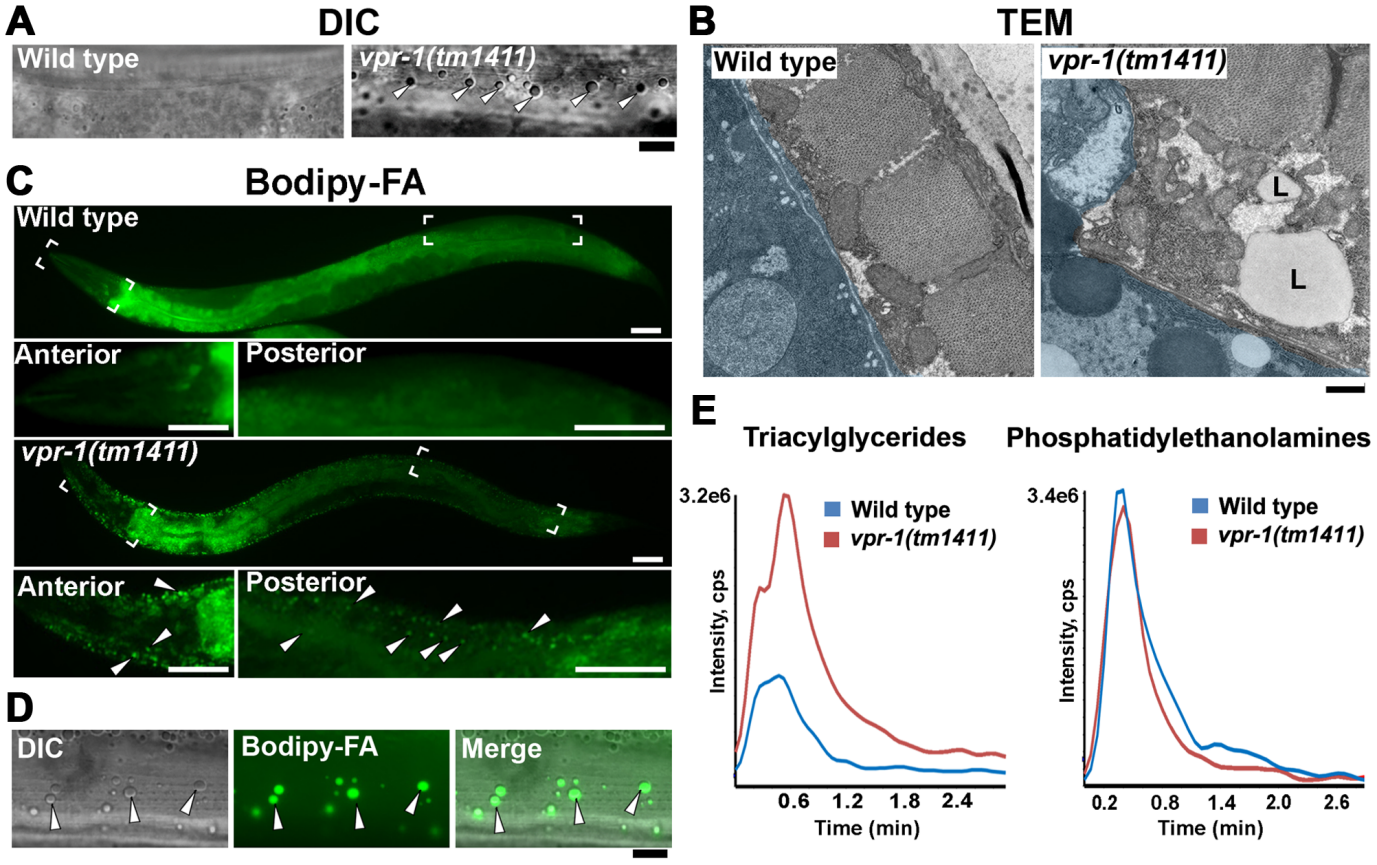
Fat levels in body wall muscle of wild-type and *vpr-1* mutant worms. (A) DIC images of muscle in live adult hermaphrodites. Arrowheads indicate lipid-like droplets. Bar, 5 µm. (B) Transmission electron micrographs of body wall muscle cytoplasm in wild-type and *vpr-1(tm1411)* mutant hermaphrodites. Light blue color demarcates muscle boundary. L, Lipid-like droplet. Bar, 0.5 µm. (C) Fluorescent images of muscle in live adult hermaphrodites fed Bodipy-FAs. Close-up images of boxed areas are shown below. Arrowheads indicate examples of Bodipy-FA-stained droplets. Anterior is to the left in all panels. Bars, 50 µm. (D) High magnification images of muscle showing Bodipy-FA-stained fluorescent droplets and droplets observed by DIC microscopy. Bar, 5 µm. (E) Comparison of total ion chromatograms of wild-type and *vpr-1(tm1411)* mutant adults extracts for 18∶0 TAG (Neutral Loss 284) and phosphatidylethanolamine (Neutral Loss 141).

In our studies of *vpr-1(tm1411)* null mutant hermaphrodites, we noticed that body wall muscles often contain large lipid-like droplets not observed in wild-type controls. These apparent lipid-like droplets were visible in young adults (1–3 days post L4 stage) by differential interference contrast (DIC) microscopy (Figure 1A). In transgenic *vpr-1* mutants expressing mitochondrial matrix-targeted GFP (mitoGFP) in muscle, droplets are observed in the muscle belly surrounded by mitochondria (Figure S1). The vast majority of visible droplets in peripheral tissues are found in muscle. Transmission electron microscopy (TEM) of *vpr-1(tm1411)* mutant muscle shows an expanded muscle belly filled with mitochondria, as previously reported [15], and large droplets (Figures 1B and S2). The droplets are often found in close proximity to mitochondria and ER. Large muscle droplets were not observed in young adult wild-type muscle (Figures 1B and S2). However, muscle lipid droplets and abnormal mitochondria are observed in very old (18 day) wild-type adults [21], [22]. In these old worms, large lipid droplets accumulate in the muscle, intestine, and epidermis. We did not detect abnormally large droplets in young *vpr-1* mutant intestinal and epidermal tissues by TEM. Instead, intestinal and epidermal tissues looked similar to wild-type controls, although it is difficult to assess minor differences (Figure S2). Hence, muscle droplets accumulate in aging *vpr-1* mutant worms.

To directly test whether these droplets contain lipid, we fed *vpr-1* mutant worms *E. coli* incubated with Bodipy-conjugated fatty acids (Bodipy-FAs). These fluorescent compounds can be used to directly visualize fat stores in live tissue [23], [24]. In wild-type hermaphrodite controls, dietary Bodipy-FAs were observed primarily in the intestine with a few small droplets present in muscle. In contrast, muscles of *vpr-1(tm1411)* null mutants contained numerous large Bodipy-FA-stained droplets (Figure 1C). The fluorescent droplets fully overlapped with those observed in muscle by DIC microscopy (Figure 1D). Similar results are observed with Sudan Black B, which darkly stains neutral TAGs in fixed opaque worms (Figure S3). Bodipy-FAs are continuously transported from the diet, to the worm's intestinal cells, and then to the muscle, where they are tightly packed in membrane-bound vesicles. Bodipy-FAs are also incorporated into yolk lipoprotein complexes [23], which are specifically endocytosed by oocytes [25]. Although yolk accumulates in the pseudocoelom of *vpr-1* mutants (due to defective oogenesis), it is not up-taken by muscle (Figure S4). Both Bodipy-FA and Sudan Black staining show a mild increase in intestinal fat content in *vpr-1* mutants. Whether this apparent increase is due to fat accumulation or increased fat synthesis is not clear.

We also performed mass spectrometry of lipid extracts to determine the lipid composition of wild-type and *vpr-1* mutant adult hermaphrodites. Lipids were analyzed by electrospray ionization tandem mass spectrometry (ESI-MS/MS). ESI-MS/MS analysis of the extracts detected a robust increase in TAGs in *vpr-1* mutant extracts, but not in the membrane phospholipids phosphatidylethanolamine and phosphatidylcholine (Figure 1E and data not shown). These data indicate that loss of *vpr-1* causes TAG accumulation in muscle of adult hermaphrodite worms.

### Increased ER stress does not cause muscle TAG accumulation in *vpr-1/vap* mutants

VAP homologs have been implicated in ER stress pathways [13], [26], [27], which can modulate lipid metabolism and homeostasis [28]. Furthermore, mitochondrial dysfunction is sometimes associated with ER stress. We considered the possibility that increased ER stress might cause the high muscle fat levels in *vpr-1* mutants. Three lines of evidence argue against this possibility. First, an integrated *hsp-4/BiPp::gfp* ER stress reporter [29] did not show elevated stress levels in *vpr-1* mutants (Figure S5A). Second, *vpr-1* mutants are not more sensitive than wild type to tunicamycin treatment, which induces ER stress (Figure S5B). Third, RNA-mediated interference (RNAi) of *xbp-1*, an ER stress-responsive transcription factor, in *vpr-1* mutants had no effect on muscle fat levels in 3-day old adults (18.0±3.6 droplets/mm^2^ for *vpr-1(tm1411)* [n = 12] versus 17.3±3.6 droplets/mm^2^ for *vpr-1(tm1411) xbp-1 RNAi* [n = 10]; *P* = 0.28). These data indicate that increased ER stress does not cause the muscle TAG defect in *vpr-1* mutants.

### *vpr-1/vap* acts cell nonautonomously to regulate fat accumulation

*vpr-1* is ubiquitously expressed and its homologs have been implicated in regulating lipid dynamics via a cell autonomous mechanism [10],[30]–[32]. To determine in which cell type(s) VPR-1 functions to regulate muscle fat, we first used genetic mosaic analysis. Transgenic *vpr-1(tm1411)* mutant hermaphrodites were generated containing the *vpr-1* genomic locus and the lineage marker *sur-5::GFP* expressed from an extrachromosomal array [33]. In *C. elegans*, extrachromosomal arrays are spontaneously lost at low frequency during cell division, thereby generating mosaic worms. When these events occur early in development, mosaic worms can be generated with losses in neurons, body wall muscles, intestinal cells, and the germ line.

**Figure 2:**
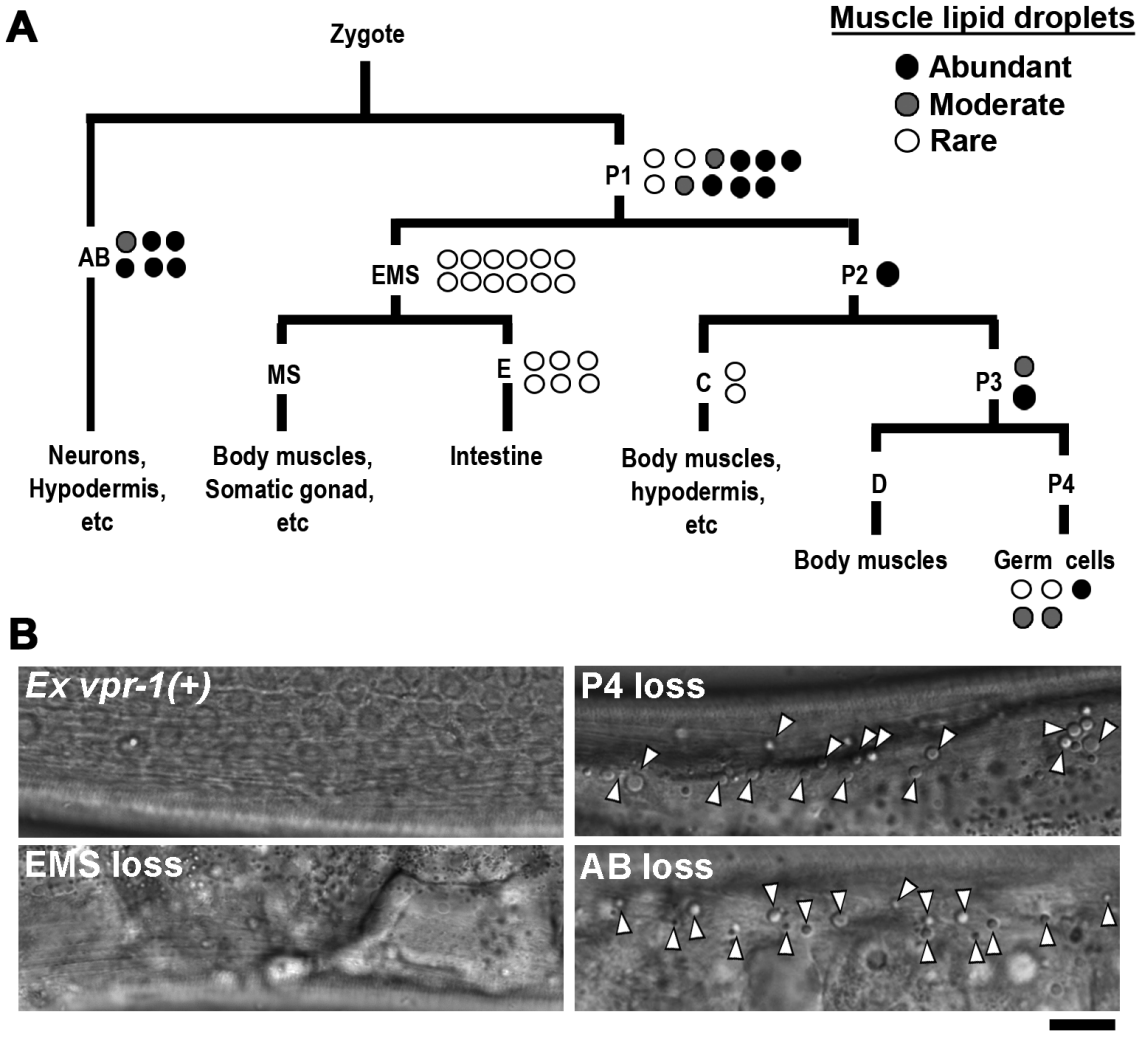
*vpr-1* mosaic analysis. (A) Analysis of vpr-1 genetic mosaics showing the lineages of major tissues. Each circle indicates one genetic mosaic worm. Points at which the genomic copy of *vpr-1(+)* was lost and the resulting phenotype are shown. (B) Representative DIC images of muscle in *vpr-1(tm1411)* mutant mosaic worms. *Ex vpr-1(+)* indicates expression of the vpr-1 genomic locus via an extrachromosomal array. Arrowheads indicate fat droplets. Bar, 5 µm.

Expressing the *vpr-1* genomic locus in *vpr-1(tm1411)* null worms rescued the fat metabolism defect in muscle (Figure 2), as well as the muscle mitochondrial defects, sterility, slow growth, and other phenotypes. Body wall muscles are generated from multiple cell lineages, including the EMS lineage. Transgene array loss in the EMS lineage generates mosaic worms that have a subset of muscles lacking *vpr-1* expression. These muscle cells exhibited low fat levels, identical to muscle cells that express *vpr-1* (Figure 2). Therefore, VPR-1 is not required in body wall muscle for fat accumulation. Mosaic worms lacking *vpr-1* in the E lineage, which generates the intestine, also did not exhibit elevated muscle fat droplets, indicating that *vpr-1* is not required in the intestine. In contrast to muscle and intestine loss, *vpr-1* loss in the AB lineage, which generates the neurons, did cause increased fat droplets in muscles (Figure 2). Unexpectedly, we also found that *vpr-1* loss in the germ cell lineage causes muscle fat accumulation (Figure 2). These results indicate that VPR-1 acts cell nonautonomously in neurons and germ cells (or their differentiation products) to modulate fat levels in muscle.

*vpr-1* null mutants are sterile, due to a failure of germ cells to differentiate into sperm and oocytes. Sperm secrete signaling molecules, such as MSPs that may influence fat metabolism [14]. To test whether sperm affect fat levels, we mated sterile 1-day-old adult *vpr-1(tm1411)* hermaphrodites to wild-type males. Supplying sperm to the reproductive tract reduces muscle fat levels in *vpr-1(tm1411)* mutants, as visualized with Bodipy-FAs (Figure S6A). Sperm did not rescue the sterility or muscle mitochondrial defects of *vpr-1* mutants (data not shown). However, preventing spermatogenesis in wild-type hermaphrodites using the *fog-3(q443)* null mutation causes mild muscle fat accumulation, as well as mild mitochondrial morphology defects (Figure S6B), without affecting oxygen consumption [34]. These data indicate that the spermatogenesis defects in *vpr-1* mutants contribute to muscle fat levels and perhaps mitochondrial defects. Two mechanisms appear to affect muscle fat levels, one mechanism involving neuronal *vpr-1* and a second mechanism involving sperm, which can modify specific *vpr-1*-dependent pathways. Here, we focus on the neuronal mechanism.

**Figure 3:**
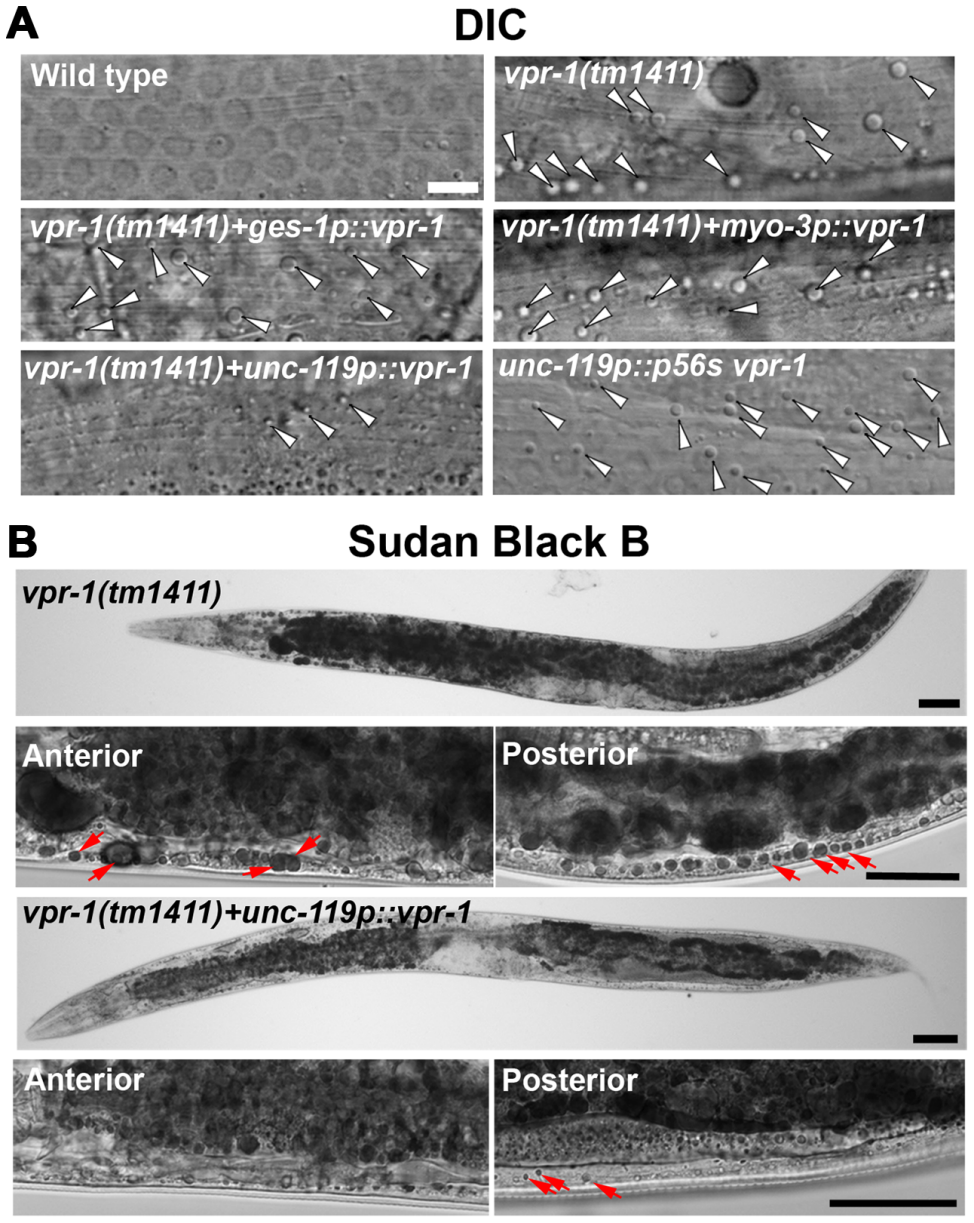
Effect of tissue-specific *vpr-1* expression on fat levels. (A) DIC images of muscle in live wild-type and *vpr-1(tm1411)* mutant hermaphrodites expressing wild-type VPR-1 or VPR-1(P56S) under indicated tissue-specific promoters. Arrowheads indicate lipid-like droplets. Bar, 5 µm. (B) Sudan Black B staining images of *vpr-1* mutants expressing *vpr-1* under the *unc-119* pan-neuronal promoter. Arrows indicate muscle fat droplets. Anterior is to the left in all panels. Wild-type controls (Figure S3) are similar to transgenic *vpr-1(tm1411)* mutants expressing *unc119p::vpr-1*. Low magnification bars, 50 µm; high magnification bars, 25 µm.

Genetic mosaics assess the effect of *vpr-1* loss from cells within an otherwise *vpr-1(+)* background. To test whether VPR-1 expression is sufficient in neurons, we expressed VPR-1 under the control of tissue-specific promoters in *vpr-1* null mutants. Consistent with genetic mosaic analysis, VPR-1 expression using the *myo-3* muscle-specific promoter or the *ges-1* intestine-specific promoter did not influence muscle fat levels. In contrast, over-expressing the *vpr-1* cDNA with the *unc-119* pan-neuronal promoter completely rescued the muscle fat levels in approximately 30–40% of transgenic mutant worms (Figures 3A and 3B). These rescued transgenic mutants were still sterile. The incomplete rescue appears to be due to the germ line defects (i.e. lack of sperm) and missing *vpr-1* introns or 3′UTR in the transgene (P. Cottee and M. Miller, unpublished). Consistent with these observations, driving neuronal expression of the *vpr-1* genomic locus instead of the cDNA rescued several *vpr-1* mutant phenotypes with increased efficiency. These results indicate that VPR-1 acts cell nonautonomously in neurons to regulate muscle fat levels.

### vMSP signaling to muscle regulates muscle fat levels

The VAPB P56S mutation acts as a dominant negative by inhibiting secretion of the wild-type and mutant vMSPs [13], [15]. To test whether neuronal vMSP secretion affects muscle fat levels, we generated transgenic worms expressing P56S VPR-1 under the *unc-119* neuronal promoter. P56S VPR-1 overexpression in wild-type worms causes increased muscle lipid droplets in most worms (Figure 3A), suggesting that vMSP secretion from neurons influences muscle fat accumulation.

**Figure 4:**
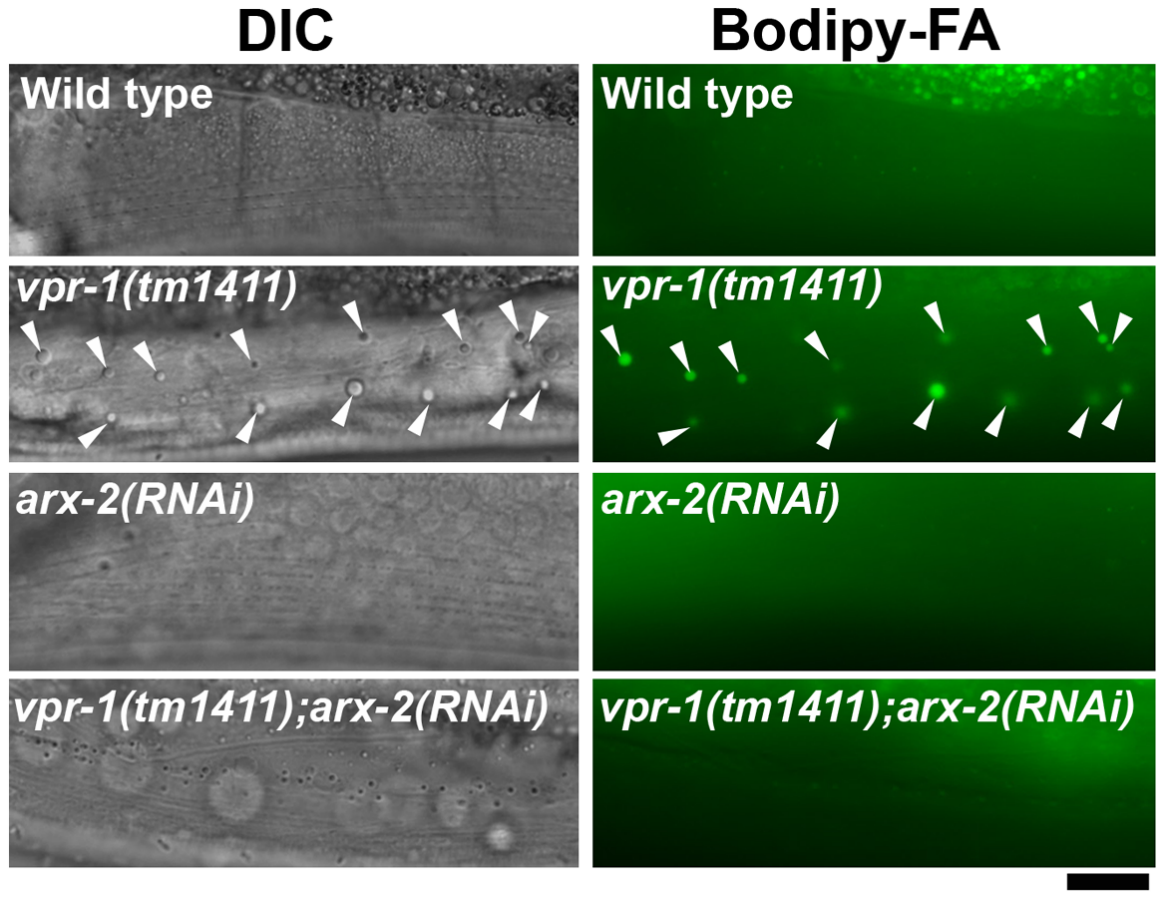
Effect of Arp2/3 inactivation on muscle fat levels. DIC and fluorescent images of muscle in live 3-day-old hermaphrodite worms fed Bodipy-FAs. *arx-2* encodes the Arp2 component of the Arp2/3 complex. Arrowheads indicate Bodipy-FA-stained fat droplets. Bar, 5 µm.

vMSP signaling to muscle is transduced via muscle SAX-3 Robo and CLR-1 Lar-like receptors [15]. *sax-3* mutations cause incompletely penetrant and variably expressed defects in muscle mitochondrial morphology [15]. Similarly, we observed incompletely penetrant defects in muscle fat accumulation by TEM and DIC microscopy (Figure S2; 11.1±13.2 fat droplets/mm^2^ for *sax-3(ky123)* [n = 13] versus 0.9±1.8 droplets/mm^2^ for wild type [n = 8]). Impaired vMSP signaling causes uncontrolled CLR-1 Lar receptor activity and ectopic Arp2/3-dependent actin filaments in muscle. A reduction of *clr-1* or *arx-2*, which encodes Arp2, rescues the muscle mitochondrial defects, but not the sterility in *vpr-1* mutants [15]. To test whether excess CLR-1 Lar and Arp2/3 activities cause muscle lipid accumulation, we used RNAi to down-regulate their functions in *vpr-1* mutants. *clr-1* or *arx-2* RNAi restored mitochondria to I-bands, as previously reported [15], and reduced muscle fat droplets in *vpr-1(tm1411)* mutants when compared to the mutant control (Figure 4; 18.0±3.6 droplets/mm^2^ for *vpr-1(tm1411)* [n = 12] versus 0.6±1.3 droplets/mm^2^ for *vpr-1(tm1411) clr-1 RNAi* [n = 9, P<0.001] and 3.1±2.0 droplets/mm^2^ for *vpr-1(tm1411) arx-2 RNAi* [n = 6, P<0.001]). Similar results were observed using TEM [15]. We also found that overexpressing *arx-2/arp2* specifically in wild-type muscle causes mild mitochondrial morphology and fat accumulation defects (Figure S7). Taken together, the data strongly support the hypothesis that impaired vMSP signaling from neurons to muscle causes elevated fat levels in muscle.

### DAF-16/FoxO is required for fat accumulation in *vpr-1/vap* mutants

**Figure 5:**
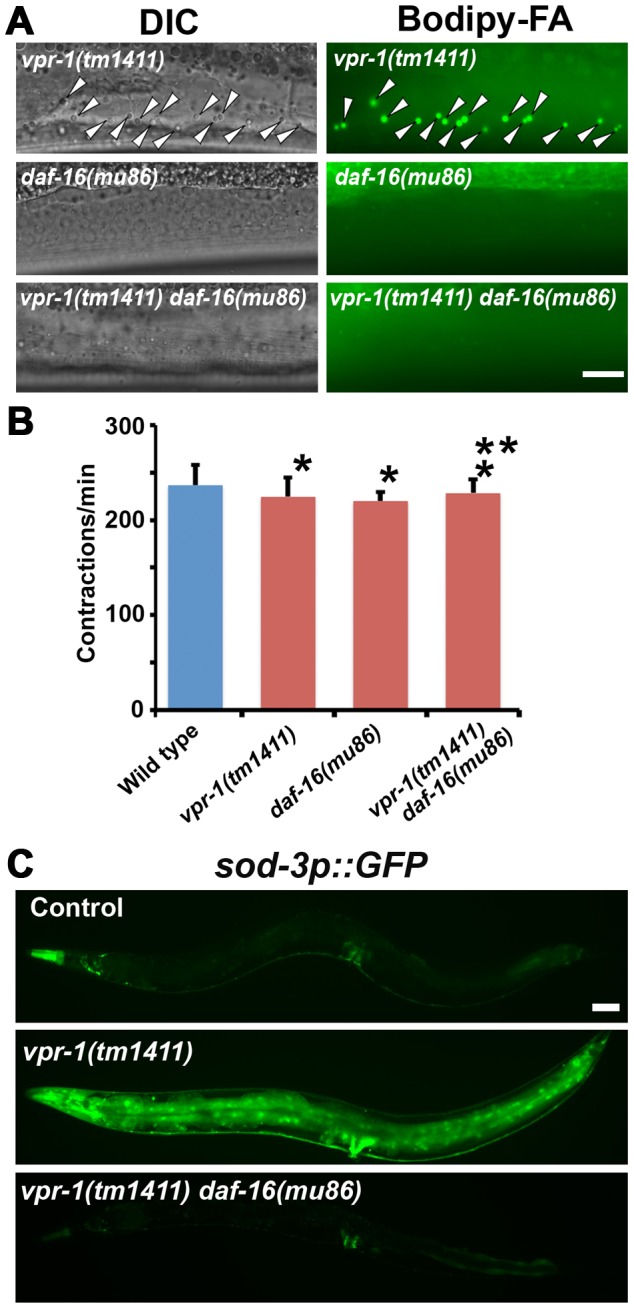
DAF-16 activity in *vpr-1* mutants. (A) DIC and fluorescent images of muscle in live 3-day-old hermaphrodite worms fed Bodipy-FAs. Arrowheads indicate Bodipy-FA-stained droplets. Wild-type controls (not shown) are similar to *daf-16(mu86)* mutants (See figures 1C and 4). Bar, 5 µm. (B) Pharyngeal pumping rates of 1-day-old adult hermaphrodites. Wild type (236.7±21.1 [n = 11]) and *vpr-1(tm1411)* mutants (220.1±9.6 [n = 11]) have similar pharyngeal pumping rate. Error bars represent SD. *P>0.05 compared to wild type. **P>0.05 compared to *vpr-1(tm1411)* mutant. (C) Transgenic worms expressing GFP under control of the *sod-3* promoter, a direct DAF-16/FoxO target. Anterior is to the left in all panels. Bar, 50 µm.

The elevated TAGs in *vpr-1* mutants and continuous accumulation of dietary Bodipy-FAs in muscle suggested that fat metabolism and transport pathways are altered. Reduced energy production triggers enhanced activity of the DAF-16/FoxO transcription factor, which controls expression of genes involved in fat synthesis, fat transport, β-oxidation, and stress resistance [35]–[39]. We hypothesized that the muscle cytoskeletal or mitochondrial defects trigger elevated FoxO activity. To investigate if DAF-16 affects fat metabolism in *vpr-1* mutants, we generated *vpr-1(tm1411) daf-16(mu86)* double mutants. Muscles of *daf-16(mu86)* null mutants contain few Bodipy-FA-stained droplets, similar to muscles of wild-type controls. However, muscle fat levels in the double mutants are also low, and strongly reduced when compared to those in *vpr-1(tm1411)* mutants alone (Figure 5A). *daf-16* loss did not affect food intake, assessed by measuring pharyngeal pumping rates (Figure 5B; *P*>0.05), muscle mitochondria (see below), or sterility of *vpr-1(tm1411)* mutants. We conclude that the elevated fat levels in *vpr-1* null mutants require DAF-16/FoxO activity.

We next examined DAF-16/FoxO transcriptional activity using an integrated transgenic line that expresses GFP under the *sod-3* promoter (*sod-3p::GFP*), a direct DAF-16 target [35], [40]. When worms were cultured under normal growth conditions, about 40–50% of 1-day-old adult *vpr-1(tm1411)* transgenic worms showed increased GFP expression relative to control transgenic animals (Figure 5C). By day three of adulthood, most *vpr-1(tm1411)* mutants show broad GFP expression throughout the body, including the intestine, neurons, vulva muscles, and body wall muscles. The elevated GFP expression is due to DAF-16 because GFP expression is suppressed in transgenic *vpr-1(tm1411) daf-16(mu86)* double mutants (Figure 5C). These data indicate that *vpr-1* loss causes elevated DAF-16 activity in muscles and other cell types.

**Figure 6:**
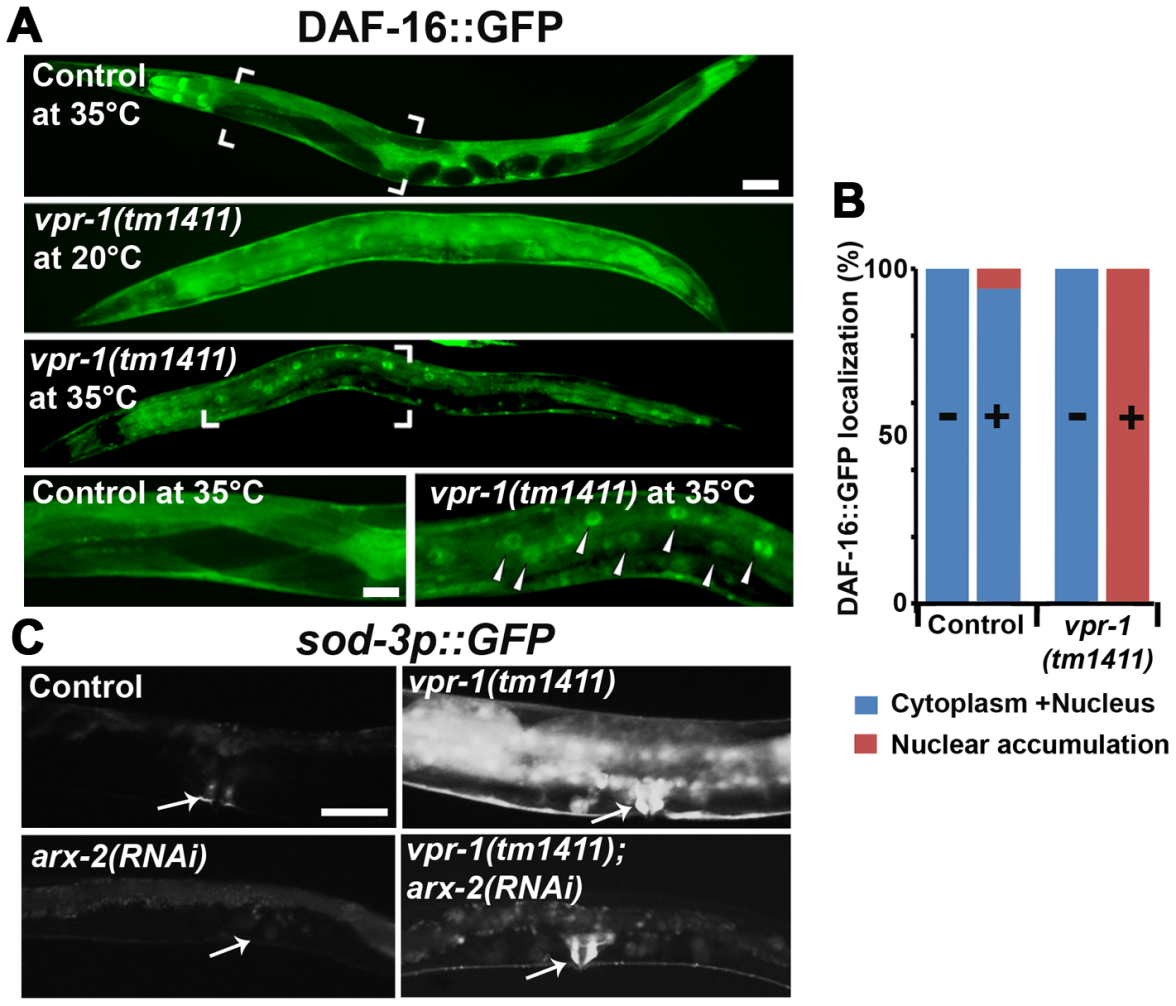
DAF-16 localization and activity in wild-type and mutant worms. (A) Transgenic strains expressing DAF-16::GFP under its endogenous promoter. Transgenic controls raised at 20°C are similar to those raised at 20°C then shifted to 35°C for 30 minutes (see panel B for quantification). Close up images of boxed areas are shown. Anterior is to the left in all panels. Low magnification bar, 50 µm; high magnification bar, 25 µm. (B) Quantification of DAF-16::GFP localization in control (n = 157) and *vpr-1(tm1411)* mutants (n = 49). (−), incubation under normal growth condition; (+), incubation at 35°C for 30 minutes. (C) Magnified images showing transgenic lines expressing GFP under the *sod-3* promoter. *arx-2* encodes Arp2. Arrows indicate vulva muscle region. Anterior is to the left in all panels. Bar, 50 µm.

To investigate the mechanism(s) by which VPR-1 controls DAF-16/FoxO, we analyzed DAF-16 subcellular localization in *vpr-1(tm1411)* mutants. An integrated and rescuing transgenic line was used to express DAF-16::GFP under its endogenous promoter. DAF-16::GFP translocates from cytoplasm to nucleus upon loss of insulin signaling, although other mechanisms exist that regulate nuclear DAF-16 activity independent of translocation [41], [42]. Under normal growth conditions at 20°C, DAF-16::GFP in *vpr-1* mutant and control transgenic strains was distributed throughout the cytoplasm and nucleus with no significant difference between the two strains (Figures 6A and 6B). However, *vpr-1* mutants appear more sensitive to higher temperatures that require increased metabolic activity (Figures 6A and 6B). We conclude that VPR-1 does not have a strong effect on DAF-16 nuclear translocation under standard conditions.

### DAF-16/FoxO likely acts downstream of the Arp2/3 complex

The results thus far strongly support the model that impaired vMSP signaling to muscle triggers DAF-16-dependent muscle fat accumulation. We hypothesized that cytoskeletal or mitochondrial abnormalities in *vpr-1* mutant muscles induce elevated DAF-16 transcriptional activity. If this idea is correct, then inactivating the Arp2/3 complex in *vpr-1* mutants should attenuate DAF-16 activity. To assess DAF-16 transcriptional activity, we used the integrated *sod-3p::GFP* transgenic reporter. *arx-2/arp2* RNAi in *vpr-1(tm1411)* mutants causes a strong reduction in *sod-3p::GFP* expression in body wall muscle, the intestine, and other cells (Figure 6C). *arx-2* RNAi in wild-type worms has little effect on GFP expression. Therefore, the elevated DAF-16 activity in *vpr-1* mutants is at least partially dependent on the Arp2/3 complex.

**Figure 7:**
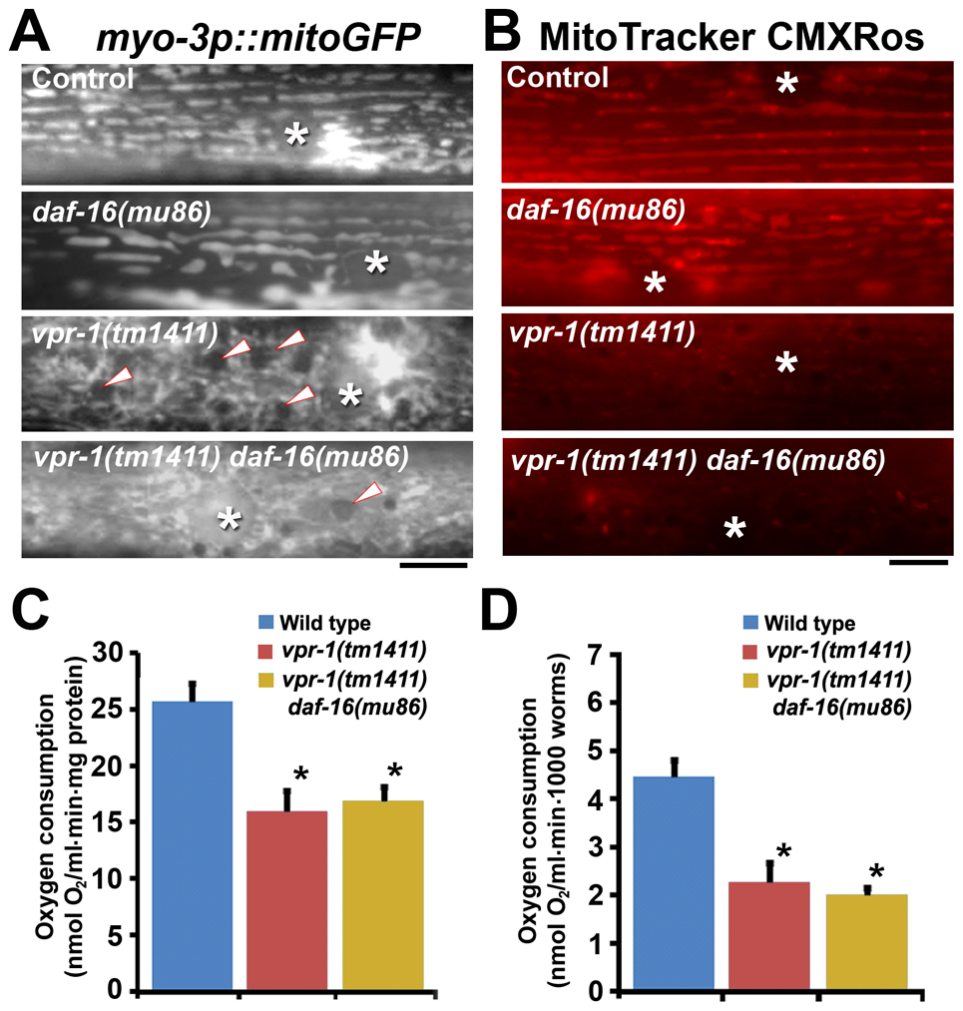
Effect of DAF-16 inactivation on muscle mitochondria. (A) Muscle mitochondrial tubules in indicated genotypes visualized using mitoGFP. Arrowheads indicate fat droplets. Asterisks indicate nucleus. Bar, 5 µm. (B) MitoTracker CMXRos staining of wild-type and mutant muscle. Asterisks indicate nucleus. Bar, 5 µm. (C and D) Oxygen consumption rates of wild-type and mutant hermaphrodites. Measured consumption rates were normalized by protein content (C) or number of worms (D). Error bars represent SD. *, *P*<0.001 compared to wild type. Oxygen consumption rate of wild-type and *vpr-1(tm1411)* mutants includes published data [15] measured together with *vpr-1(tm1411) daf-16(mu86)* mutants.

One possibility is that DAF-16 causes the mitochondrial abnormalities in *vpr-1* mutants. To test this model, we first evaluated mitochondria using the mitoGFP transgene expressed in body wall muscle. As previously documented [15], wild-type muscles contain linear mitochondrial tubules positioned along I-bands. In contrast, *vpr-1(tm1411)* mutants contain disorganized and interconnected mitochondrial networks in the muscle belly (Figure 7A). Loss of *daf-16* in *vpr-1(tm1411)* mutants did not affect muscle mitochondrial morphology or localization (Figure 7A). Next, we examined mitochondrial functional status using MitoTracker CMXRos, which accumulates in the mitochondrial matrix depending on membrane potential, and oxygen consumption of whole worms. DAF-16 loss did not affect the reduced MitoTracker CMXRos accumulation (Figure 7B) or the low oxygen consumption rates of *vpr-1* mutants (Figures 7C and 7D). We conclude that DAF-16 does not affect the muscle mitochondrial defects in *vpr-1* mutants and likely acts downstream of Arp2/3.

### DAF-16/FoxO increases ATP levels and extends lifespan of *vpr-1/vap* mutants

**Figure 8:**
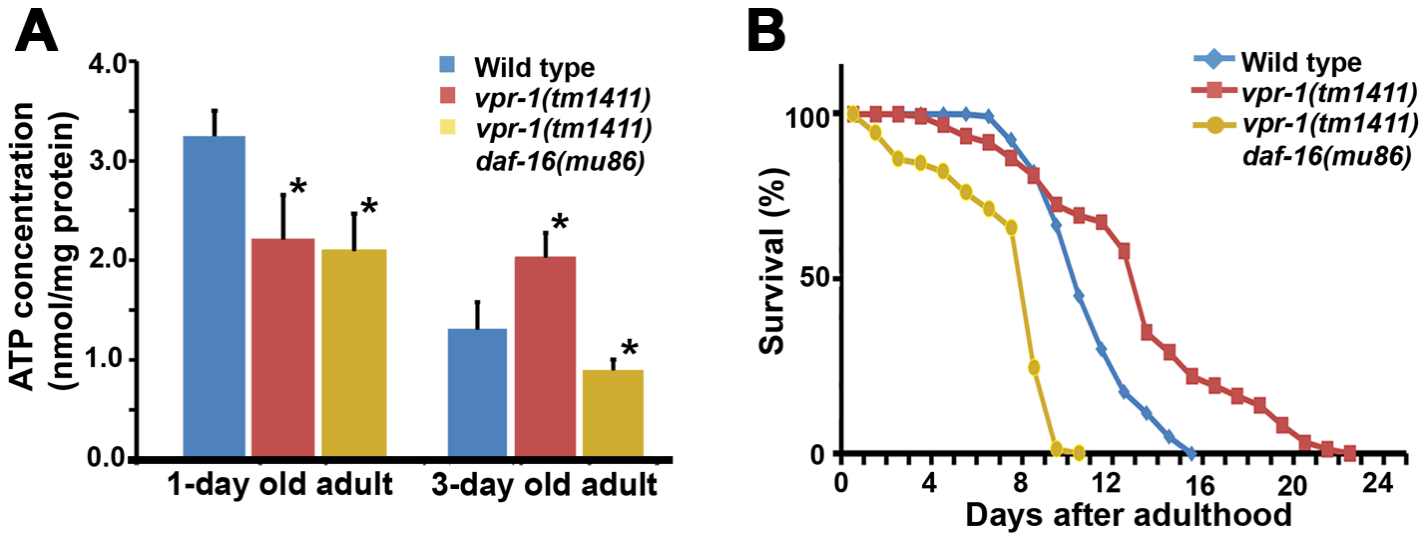
Effect of DAF-16 inactivation on ATP level and lifespan. (A) ATP concentration in wild-type and *vpr-1(tm1411)* mutant adult extracts. *, *P*<0.001 compared to wild type. Error bars represent SD. ATP concentration of wild-type and *vpr-1(tm1411)* mutants at 1-day-old adults include published data [15] measured together with *vpr-1(tm1411) daf-16(mu86)* mutants. (B) Lifespan measurements of indicated genotypes. The lifespan of *daf-16(mu86)* mutants (not shown) was similar to the wild type, as previously shown.

As the intestine and epidermis are fat storage sites in *C. elegans*, we hypothesized that the increase in muscle fat is an attempt to provide fuel for energy production. Our previous studies showed that 1-day-old adult *vpr-1(tm1411)* mutants have reduced ATP levels when compared to controls [15]. However, the ATP levels in *vpr-1* mutants did not decrease over the next two days, as observed in the wild type (Figure 8A). 3-day-old adult *vpr-1(tm1411)* mutants had higher ATP levels than wild-type controls at the same age (Figure 8A). Similar ATP dynamics have been observed in aging worms with mutations in the *daf-2* insulin receptor or *clk-1*, a mitochondrial protein involved in ubiquinone biosynthesis [43], [44]. Hence, DAF-16 may help maintain ATP levels in these aging worms. To test whether DAF-16 affects the energy balance of *vpr-1* mutants, we measured ATP levels in single and double mutant extracts. *daf-16* loss did not influence ATP levels in 1-day-old adult *vpr-1(tm1411)* mutants (Figure 8A). However, *daf-16* is required for the high ATP concentration in 3-day old mutant adults (Figure 8A; *P*<0.001). ATP levels in *daf-16* mutants are similar to wild-type controls (data not shown), as previously shown [43], [44]. These data indicate that DAF-16/FoxO helps *vpr-1* mutants maintain ATP levels during aging.

Based on the abnormalities in energy metabolism, we tested whether DAF-16 influences lifespan in *vpr-1* mutants. Similar to other worm mutants with mild or tissue-specific reduction in mitochondrial function, *vpr-1(tm1411)* mutants have slightly extended adult lifespan compared to wild-type worms (Figure 8B; mean adult lifespan ± SD of 12.9±4.4 days [n = 154] for *vpr-1(tm1411)* versus 10.5±2.1 days [n = 159] for wild type, *P*<0.001). *daf-16* loss in *vpr-1(tm1411)* mutants causes a strong reduction in lifespan relative to *vpr-1* mutants and wild-type controls (Figure 8B; 6.9±2.5 days for *vpr-1(tm1411) daf-16(mu86)* [n = 250]; *P*<0.001). The lifespan of *daf-16* single mutants was similar to wild type (data not shown), as previously shown [38], [45]. These data indicate that DAF-16/FoxO activity extends survival of *vpr-1* mutants.

### *Vapb* knockout mice exhibit signs of abnormal skeletal muscle energy metabolism

**Figure 9:**
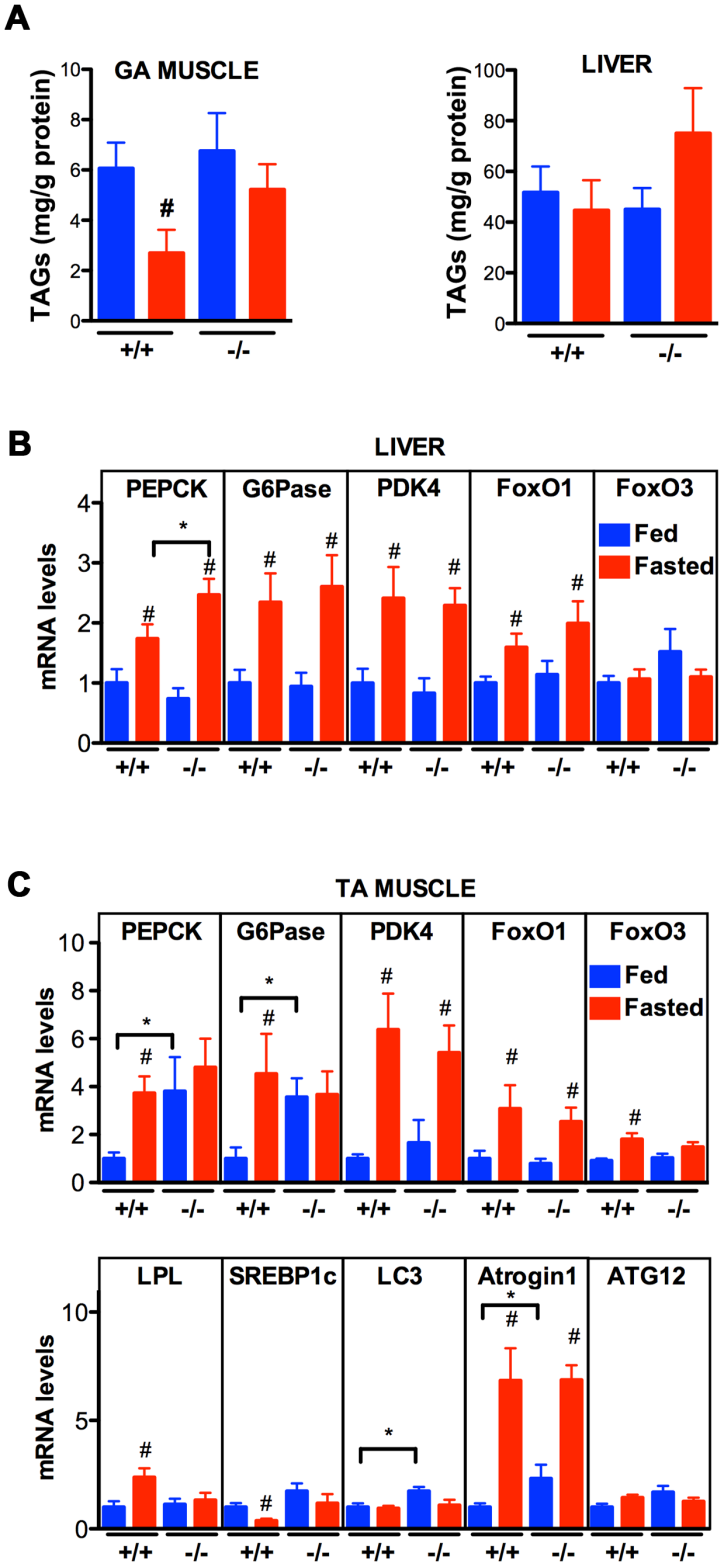
Effect of *Vapb* ablation on fasting/refeeding energy metabolism in mice. (A) TAG concentration in GA muscle and liver of wild-type (+/+) and *Vapb* knock-out (−/−) mice after 24-hour fasting (red) or 24 hours fasting followed by 6 hours of refeeding (blue). (B and C) Quantitative RT-PCR of indicated genes in liver (B) and TA muscle (C) of wild-type (+/+) and *Vapb* knock-out (−/−) mice after 24-hour fasting (red) or 24 hours fasting followed by 6 hours of refeeding (blue). Relative mRNA levels are shown on the Y-axis. #, *P*<0.05 compared to fed mice of the same genotype. *, *P*<0.05 compared to +/+ under the same condition.

The data thus far indicate that VPR-1 loss causes profound defects in muscle energy metabolism. We hypothesized that the regulatory function of vMSPs on energy metabolism was conserved in mammals, and studied energy metabolism of *Vapb* −/− mice [4]. In basal conditions, *Vapb −/−* mice do not exhibit overt defects in energy metabolism. In particular, body weight and glycemia appear normal with age (L. Dupuis, unpublished results). However, an energy metabolism defect of *Vapb* deficient mice might be unmasked by modifying insulin supply through feeding and fasting paradigms. In worms and mice, fasting reduces insulin signaling and increases FoxO activity, resulting in altered metabolic gene expression. We used *Vapb* −/− mice of 2–6 months of age to avoid any confounding effect of the motor dysfunction observed at 18 months [4]. Mice were either fasted for 24 hours (fasted group) or fasted for 16 hours and refed for 8 hours to synchronize meals (fed group). In +/+ mice, fasting decreased the TAG levels in the gastrocnemius (GA) muscle (Figure 9A; *P*<0.05). In contrast, TAG levels remained unchanged upon fasting in *Vapb −/−* GA and tibialis anterior (TA) muscles (Figure 9A and data not shown). In liver, TAG levels were unchanged upon fasting and feeding in either +/+ or −/− mice (Figure 9A). Thus, *Vapb* ablation increases the resistance of muscle lipid stores to fasting induced mobilization.

We next looked at mRNA levels of metabolic genes by quantitative RT-PCR. In liver, *Vapb* ablation potentiated induction of the direct FoxO1 target gene phosphoenolpyruvate carboxykinase (PEPCK) in response to fasting, but had no effect on fasting induction of other FoxO1 targets such as glucose 6-phosphatase (G6Pase) and pyruvate dehydrogenase kinase (PDK4) (Figure 9B). FoxO1 and FoxO3 mRNA and proteins were similar in +/+ and −/− livers, and FoxO1 up-regulation by fasting appeared normal in −/− liver (Figure 9B).

We also examined putative FoxO1 and FoxO3 target genes in +/+ and mutant TA muscle. Feeding decreased expression of PEPCK, G6Pase, and lipoprotein lipase (LPL), and increased expression of the lipogenic transcription factor SREBP1c (Figure 9C). This regulation was lost in *Vapb* −/− muscles, as feeding did not modify expression of these four genes. *Vapb* genotype did not affect levels of PDK4 mRNA. FoxO1 and FoxO3 expression was down-regulated upon feeding in control TA muscles, but FoxO3 regulation was lost in −/− muscles (Figure 9C). The expression of muscle FoxO3 targets LC3 and Atrogin1 was up-regulated in fed −/− mice, while another FoxO3 target, ATG12, was unchanged. These results indicate that muscles of *Vapb* −/− mice are partially insensitive to fasting/feeding alterations in lipid mobilization and FoxO target gene expression. Hence, *Vapb* mutant worms and mice appear to have muscle energy metabolism alterations, at least in part involving FoxO targets. Whether the putative metabolic changes in mouse muscle are due to secreted vMSPs is not yet clear.

## Discussion

Results from Drosophila and *C. elegans* support the model that VAP MSP domains are secreted neurogenic factors that promote striated muscle oxidative metabolism [15]. In *C. elegans*, neurons cleave the vMSP and secrete it into the surrounding environment. Secreted vMSPs signal through SAX-3 Roundabout and CLR-1 Lar-like receptors expressed in muscle, down-regulating Lar signaling to the Arp2/3 complex. This signaling pathway restricts actin filament formation to I-bands of the myofilaments, thereby localizing mitochondria to I-bands and promoting mitochondrial function [13], [15]. Here we show that impaired vMSP signaling to muscle triggers increased DAF-16/FoxO transcription factor activity. FoxO promotes TAG accumulation in muscle, helps maintain ATP levels during aging, and extends lifespan. We propose that reduced vMSP signaling puts animals in an energy deficit, which triggers an altered metabolic response involving FoxO. Evidence for this model and implications for ALS are discussed below.

### A VAPB cell nonautonomous mechanism for regulating muscle TAGs

VAPs physically interact with multiple proteins involved in lipid binding and transport, such as oxysterol binding protein and ceramide-transfer protein [10], [11], [30], [46]. Although the biological role of these interactions is not well understood, VAPs have been proposed to act in macromolecular complexes for transporting lipids between organelles at membrane contact sites. This mechanism depends on VAP function in the same cell in which lipid dynamics occur (i.e. a cell autonomous function). Here we show in *C. elegans* that *vpr-1/vap* loss triggers a robust increase in striated muscle TAG levels. Unexpectedly, this function does not require VPR-1 in muscle. Genetic mosaic and cell-type specific expression studies demonstrate that VPR-1 acts in neurons, consistent with the signaling function. Indeed, muscle vMSP receptors and the downstream Arp2/3 complex mediate this lipid metabolism response. We also found that sperm presence can modulate striated muscle TAG metabolism. Neurons and sperm are two cell types capable of secreting MSP domains [15], [47]. Our data do not exclude cell autonomous roles for VPR-1 in regulating lipid dynamics. Nevertheless, they highlight the importance of testing VAP autonomy when evaluating biological mechanism.

### The connection between VAPB and FoxO

We show that *vpr-1/vap* loss triggers elevated DAF-16/FoxO activity, resulting in muscle TAG accumulation. Inactivating the Arp2/3 complex largely suppresses these metabolic alterations, as well as the muscle mitochondrial defects. These data support the model that impaired vMSP signaling to muscle triggers elevated FoxO activity. Consistent with this model, over-expressing Arp2 specifically in wild-type muscle causes TAG accumulation and mitochondrial defects. Although we cannot eliminate the possibility that Arp2/3 acts in other tissues, it appears to be a muscle-specific suppressor of *vpr-1* mutants. How might the Arp2/3 complex regulate FoxO? One possibility is that *vpr-1* mutants go into energy deficit as they age, as mitochondrial dysfunction is thought to increase FoxO activity [48]–[51]. An alternative possibility is that FoxO acts downstream of Arp2/3, but in parallel to mitochondria. In either case, reduced insulin signaling could be involved. A strong reduction in insulin causes increased FoxO nuclear translocation, which is not observed in *vpr-1* mutants under standard conditions. However, subtle changes can be more difficult to detect.

Additional mechanisms could also modulate FoxO in *vpr-1* mutants. The vMSP/ephrin receptor VAB-1 directly interacts with DAF-18/PTEN (phosphatase and tensin homolog deleted on chromosome ten), which regulates FoxO activity [52]. VAB-1 is expressed throughout the adult nervous system and in the gonad [53], [54]. Previous studies have shown that sperm presence can modulate DAF-16/FoxO translocation and transcriptional activity [55], perhaps through secreted MSPs. Whether sperm act via the Arp2/3 complex is not clear. An interesting possibility is that global MSP signals from neurons and sperm are sensed through distinct mechanisms. These mechanisms might converge on muscle metabolic output to meet changes in energy requirements.

In mammals, FoxO transcription factors are critical regulators of energy metabolism, particularly under fasting conditions. We show that *Vapb* ablation in mice renders muscle lipid stores resistant to fasting, a situation analogous to lipid accumulation in *vpr-1* mutant worm muscles. Dysregulated lipid stores in mutant mice is associated with alterations in muscle gene expression consistent with abnormal FoxO1 and FoxO3 activity [56]. For instance, FoxO1 target gene mRNAs for PEPCK and G6Pase are clearly up-regulated in muscle of young *Vapb* −/− mice in the fed state (*i.e.* in the presence of insulin that decreases FoxO1 activity). Similar results are observed for FoxO3 target genes LC3 and Atrogin-1. These data suggest that FoxO1/3 are less sensitive to insulin inhibition in *Vapb* −/− mice.

Not all FoxO target genes studied are sensitive to *Vapb* ablation. For instance, VAPB does not appear to influence PDK4 and ATG12 mRNAs. Additionally, some of the mRNAs studied showed uncoupling from circulating insulin levels, consistent with an insensitivity of FoxO1 to insulin. SREBP1c mRNA, which is negatively regulated by FoxO1 [57], was increased by feeding in +/+ mice, but not in −/− mice. A similar, albeit mirror situation was observed for LPL, a gene positively regulated by FoxO1 [58]. Hence, FoxO1/3 might participate in the abnormal lipid mobilization in *Vapb* −/− mice, but other mechanisms are likely at work to avoid the major consequences of chronic muscle FoxO activation, such as muscle atrophy [59]. In summary, our findings show that VAPB is involved in modulating mouse muscle energy metabolism upon fasting and refeeding, possibly via altered FoxO activity. Whether this occurs through a cell autonomous or a cell nonautonomous mechanism, like in *C. elegans* and Drosophila, remains to be determined.

### FoxO is protective in *vap* mutants

A key finding in worms is that DAF-16/FoxO activity prolongs the adult lifespan of *vpr-1* mutants from 6.9±2.5 to 12.9±4.4 days. This lifespan increase may be due to metabolic alterations that compensate for mitochondrial dysfunction. Consistent with this idea, FoxO extends the lifespan of *C. elegans* with reduced mitochondrial function [48], [49], [60]. The FoxO-dependent fat accumulation in *vpr-1* mutant muscle may reflect an effort to increase energy production. We show that DAF-16 helps *vpr-1* mutants maintain ATP levels in 3-day old adults. Among the numerous DAF-16 metabolic genes are those involved in fat synthesis and transport, β-oxidation, the glyoxylate cycle, and gluconeogenesis [37]. However, additional DAF-16 targets may also be involved, such as stress resistance enzymes [37], [38], [61]. *vpr-1* mutants are more resistant than the wild type to reactive oxygen species and ER stress. Based on identified DAF-16 targets and *vpr-1* mutant phenotypes, DAF-16 might increase energy substrate availability in muscle, stimulate anaerobic metabolism, increase oxidative metabolism in non-muscle cells, or decrease ATP consumption. Further studies are necessary to distinguish among these possibilities, as well as other models.

### Implications for ALS

Metabolic alterations in ALS patients and mouse models are hypothesized to compensate for mitochondrial dysfunction, particularly in skeletal muscle [17], [19], [62], [63]. Differentially expressed gene networks involved in oxidative metabolism and the cytoskeleton, including up-regulated FoxO1 and FoxO3 mRNAs have been found in ALS patient skeletal muscles [64], [65]. Our studies of VAPB in worms, flies, and mice are consistent with impaired vMSP signaling to muscle causing some of these alterations. Importantly, *vpr-1* loss in worms, *Vapb* depletion in zebrafish, or *Vapb* loss in mice does not cause motor neuron degeneration [4], [15], providing strong evidence that mitochondrial and metabolic defects are not secondary consequences of neurodegeneration. These data contrast with a recent Drosophila study suggesting that *Vapb* loss causes neurodegeneration via increased phosphoinositides [66]. In humans, metabolic alterations caused by reduced VAPB function may not be sufficient to induce motor neuron degeneration, although they could strongly predispose to ALS. Redundancy could be an important consideration in the different models. The worm genome encodes a single *vap* homolog, but many genes with MSP domains. Vertebrate genomes typically encode VAPA and VAPB, which are approximately 60% identical. *Vap* mutant flies have the most severe developmental defects and the fewest MSP genes in the genome.

In summary, our results support the model that striated muscle mitochondrial dysfunction alters FoxO activity, which in turn affects energy metabolism and promotes survival. It is possible that reduced vMSP signaling causes some of the mitochondrial and metabolic alterations in ALS patients. Perhaps vMSPs might protect against ALS via effects on skeletal muscle energy metabolism.

## Materials and Methods

### *C. elegans* genetics, strains, and RNA-mediated interference

*C. elegans* Bristol N2 is the wild-type strain. Worms were grown on NGM plates with NA22 bacteria as the food source [67]. Strain construction and marker scoring were done as previously described [15], [54]. The strains and genetic markers used or generated were as follows: CF1553 muIs84[pAD76(*sod-3::GFP*)], CF1038 *daf-16(mu86)* I, *vpr-1(tm1411)/*hT2[*bli-4*(*e937*) let-?(*q782*) qIs48] I;III, SJ4005 zcIs4[*hsp-4::GFP*], TJ356 zIs356[*daf-16p::daf-16::GFP; rol-6*] IV, *fog-3(q443) I/*hT2[*bli-4*(*e937*) let-?(*q782*) qIs48] I;III, CX3198 *sax-3(ky123) X*, and XM1004 *vpr-1(tm1411) daf-16(mu86)/*hT2[*bli-4*(*e937*) let-?(*q782*) qIs48] I;III. Transgenics expressing *vit-2p::vit-2::gfp* were generated by crossing into the pwIs23 integrated line. RNAi was performed using the feeding method starting at the L1 stage, as previously described [15]. *arx-2*, *clr-1*, and *xbp-1* RNAi clones are from the genome-wide library [68]. Each clone was sequenced for confirmation.

### Transgenics

To generate transgenic *C. elegans*, the marker plasmids pRF4 [*rol-6*] (60 ng/µl) or *myo-3p::mito::GFP* (30–60 ng/µl) were mixed with *myo-3p::vpr-1* (60 ng/µl), *ges-1p::vpr-1*(60 ng/µl), *unc-119p::vpr-1*(60 ng/µl), *unc-119p::vpr-1* P56S (60 ng/µl), or *myo-3p::arx-2::mCherry* (60 ng/µl) and microinjected into the gonads of young adult hermaphrodites. Injected worms were incubated for 24 hours, transferred to new NGM plates, and screened for transgenic progeny. Transgenic lines were selected based on the roller phenotype or GFP expression. Multiple independent transgenic lines were generated for all strains. To conduct genetic mosaic analysis, 10 ng/µl WRM06B28 fosmid DNA containing the *vpr-1* genomic locus was mixed with 10 ng/µl pTG96 (*sur-5p::GFP*) plasmid and microinjected into the gonads of *vpr-1(tm1411)*/hT2 hermaphrodites. Transgenic lines were selected based on GFP expression. Transgenic lines were maintained as *vpr-1(tm1411)* homozygotes, as the fosmid rescued the sterility, mitochondria, fat metabolism, slow growth, and embryonic defects. For lineage scoring, approximately 15,000 worms were screened. Transgene loss in the AB lineage was scored by GFP loss in head and tail neurons, the nerve cords, and the excretory gland. Transgene loss in the P1 lineage was scored by GFP loss in the intestine, muscle, somatic gonad, and hyp11. The P2 lineage was scored by GFP loss in numerous body wall muscle cells and hyp11, the P3 lineage was scored by GFP loss in body wall muscle, and the P4 lineage was inferred by a sterile phenotype without GFP loss. Transgene loss in the EMS lineage was scored by GFP loss in the intestine and somatic gonad, while loss in the E lineage was score by exclusive GFP loss in the intestine.

### Transmission electron microscopy

TEM was performed as previously described [15].

### Bodipy-FA and Sudan Black B staining

For the Bodipy-FA experiments, a 5 mM Bodipy-FA (Molecular probe, U.S.A) stock solution was prepared in DMSO and kept at −20°C. A 200 µM working solution diluted in distilled water was dropped onto seeded plates and allowed to dry. L4 stage worms were placed on the plates and incubated in the dark for 24 hours at 20°C. Bodipy-FAs can get trapped in intestinal gut granules that are not present in muscle.

Sudan Black B staining was conducted as described in previous studies [15]. Briefly, synchronized 1-day-old adult worms were collected into microfuge tubes containing M9 solution. Worms were washed five times, incubated for 40 minutes at 20°C to remove intestinal bacteria, and fixed in 1% paraformaldehyde. The fixed worms were washed three times in cold M9 solution and dehydrated through a 25%, 50%, and 70% ethanol series. Sudan Black B solution was added to the worms and incubated for 1 hour. To remove excess stain, worms were washed five times with 70% ethanol. To normalize for staining variability among experiments, wild type and *vpr-1(tm1411)* mutants were processed in the same tube and identified based on gonad morphology.

### Lipid analysis by ESI-MS/MS

For the lipid analysis by ESI-MS/MS, lipids from equal masses of wild type and *vpr-1(tm1411)* mutant adults were extracted by chloroform-methanol following a modified Bligh/Dyer extraction [69]. A mixture of internal standards including T17:1 TAG was added to the chloroform-methanol phase before extraction. The extracted samples were concentrated to dryness under a nitrogen stream, reconstituted with methanol∶chloroform (1∶1 v/v) and transferred to HPLC auto samplers. Lipids were analyzed by ESI-MS/MS using an API 4000 (Applied Biosystems/MDS Sciex, Concord, Ontario, Canada) triple quadrupole mass spectrometer. Extracted lipid samples (5 ml) were infused into the mass spectrometer with a solvent mixture of chloroform-methanol (1∶2, v/v) containing 0.1% formic acid using a Shimadzu Prominence HPLC with a refrigerated auto sampler (Shimadzu Scientific Instruments, Inc. Columbia, MD). Lipids were analyzed in positive ion mode using an API 4000 (Applied Biosystems/MDS Sciex, Concord, Ontario, Canada) triple quadruple mass spectrometer. Samples (5 µl) were directly infused into the electrospray source using a Shimadzu Prominence HPLC with a refrigerated auto sampler (Shimadzu Scientific Instruments, Inc. Columbia, MD). Neutral loss (NL) scanning (228, 254, 256, 268, 278, 280, 284, and 304) of naturally occurring aliphatic chains (i.e. building block of TAG molecular species) were utilized to determine the identities of each molecular species. NL scanning of 141 was used for profiling phosphatidylethanolamine. The following analysis parameters were used: ion spray voltage 5000 V, de-clustering potential 40 V, temperature 300°C (for TAG), collision energy 35 V, and collisionally activated dissociation 5.

### Mitochondrial staining

To assess mitochondrial transmembrane potential, worms were stained using the MitoTracker CMXRos dye (Molecular Probes, U.S.A), as previously described [15]. This lipophilic cationic fluorescent dye accumulates in mitochondria in a membrane potential-dependent manner [70]. L4 larval stage worms were placed on dried plates containing a 100 µM MitoTracker CMXRos dye solution (dropped on bacteria). After 24 hours incubation in the dark, worms were transferred to a new NGM plate and incubated in the dark for 20 minutes to remove intestinal background. Worms were mounted on dried 2% agarose pads without anesthetic. Wild-type and *vpr-1(tm1411)* mutant hermaphrodites were cultured on the same plates.

### ATP concentration measurement

ATP concentration was measured as described previously, with slight modification [15]. Briefly, 150 worms were individually picked and placed into tubes containing M9 buffer, washed four times, and incubated at 20°C for 40 minutes to remove intestinal bacteria. These worms were then washed four times with TE solution (100 mM Tris–Cl, pH 7.6, 4 mM EDTA) and placed into microfuge tubes containing 300 µl TE solution. Worm extracts were prepared by a series of cycles including freezing, thawing, and sonicating. These extracts were boiled for 10 minutes to release ATP and block ATPase activity. Carcasses and insoluble material were pelleted in a microcentrifuge at 20,000×g for 10 minutes. The soluble extracts were diluted in a 1∶10 ratio using TE solution. ATP concentration in 60 µl of diluted extracts was measured using the ENLITEN ATP Assay System (Promega, U.S.A), according to the manufacturer's instructions. A luminometer (Berthold, Germany) was used for quantification. Protein concentration was determined using the BCA protein assay (Pierce, U.S.A). ATP measurements were repeated at least three times for each strain.

### Oxygen consumption

Oxygen consumption rates were measured as previously described using the oxygraph system (Hansatech, UK) with minor modifications [15]. Worms were cultured at 20°C and synchronized to the 1-day-old adult stage. For each test, 1000 worms were individually picked and placed into a glass tube with 1 ml M9 buffer at 20°C. Collected worms were incubated for 40 min at 20°C to remove intestinal bacteria, carefully washed five times, and placed into 1 ml M9 buffer. The worm solution was loaded into the chamber equipped with a S1 Clark type polarographic oxygen electrode disc maintained at 20°C. Oxygen concentration was measured for 10 minutes. For normalization, worms were carefully collected from the chamber and protein content was measured using the BCA test kit (Pierce, U.S.A.). Rates were normalized to either total protein content or number of worms. We performed at least three independent measurements per strain.

### Feeding rate and lifespan assays

To measure feeding rates, worms were cultured at 20°C and 1-day-old adult worms were placed on new NGM plates. Feeding behavior was recorded using a Zeiss Lumar stereomicroscope with AxioCam MRM digital camera. Measurements were conducted during a 30 second period at room temperature (22°C). The rhythmic contractions of the pharyngeal bulb were counted. For each strain, over 20 worms were counted. To determine lifespan of worms, L4 larval stage worms were placed on new NGM plates seeded with NA22 bacteria and cultured at 20°C. The L4 stage was used because a small percentage of *vpr-1* mutants die during L1–L4 stages and *vpr-1* mutants develop slowly. Worms were monitored every day and transferred to flesh NGM plates. Death was scored by failure to respond to touching with a platinum wire. Wild-type worms fed NA22 bacteria have slightly shorter lifespan than worms fed OP50 bacteria.

### Tunicamycin resistance

To analyze ER homeostasis, worms were cultured on plates with tunicamycin (Sigma, U.S.A) from the embryonic stage to adulthood. NGM plates with 0.1% DMSO and 0 or 5.0 µg/ml tunicamycin were prepared. About 30 adult worms were placed on each tunicamycin plate and allowed to lay embryos for 30 minutes. Adult worms were then removed. Twelve hours later the number of hatched embryos was counted and compared with the number of worms that reached the adult stage within 96 hours. We performed at least three independent measurements for each strain.

### Mouse experiments

Mouse experiments were performed using the Institutional European Guidelines, under the supervision of an authorized investigator (LD), and approved by the local ethical committee for animal experiments (CREMEAS, agreement N° AL/01/08/02/13). *Vapb* −/− mice were used and genotyped as described [4]. Mice (8–10 per group) were either fasted for 24 hours from 5PM (fasted group), or fasted from 5PM to 9AM and refed until sacrifice at 5PM. GA and TA muscle and liver tissues were collected, and rapidly frozen in liquid nitrogen for subsequent analyses of gene expression and TAG levels. The tissues were stored at −80°C until the time of analysis.

For RT-qPCR, frozen liver and muscle tissues were placed into tubes containing 5 mm stainless steel beads (Qiagen, Courtaboeuf, France) and 1 ml of Trizol reagent (Invitrogen, Paisley, UK) and homogenized using a TissueLyser (Qiagen). RNA was prepared from tissue homogenates following Trizol manufacturer's instructions. RNA reverse transcription and SYBR Green real-time PCR assays were performed using the Bio-Rad (Biorad, Marnes la Coquette, France) iCycler kits and protocols. PCR conditions were 3 min at 94°C, followed by 40 cycles of 45 s at 94°C and 10 s at 60°C. Primers are shown in Table S1. For western blotting, liver and TA muscles were incubated in Lysis buffer containing complete protease and phosphatase inhibitor cocktails. Protein concentration was measured using BCA Protein Assay. Equal amount of protein (50 µg) were separated by SDS-PAGE 10% and blotted onto nitrocellulose membrane. Membranes were saturated with 10% milk and then incubated with the primary antibodies FoxO1 (Proteintech; 18592-1-AP), FoxO3a (Cell signaling; #2497), VAPB [4] and Histone H3 (Cell signaling; #9715), all diluted (1∶1000) followed by anti-rabbit secondary antibody, diluted 1∶5000.

For TAG analysis, tissue powder was homogenized in lysis buffer (250 mM Sucrose solution, 1 mM EDTA, 2% SDT, 1 mM DTT, 10 mM Tris HCl pH 7.4) containing protease inhibitors (Sigma P8340) and phosphatase inhibitors (Sigma 8345), centrifuged at 12000×rpm for 15 minutes at room temperature. TAG concentration was determined in duplicate for each sample in 5 µl of supernatant, using the enzymatic method of analysis (Randox *Triglyceride* Colorimetric Assay *Kit*, *Randox Laboratories Limited*, *UK*) as described by the manufacturer. Lipid values were normalized to protein concentration.

## Supporting Information

Figure S1 Lipid-like droplets and mitochondria in *vpr-1* mutant striated muscle. (A) Close-up image of a live transgenic *vpr-1(tm1441)* mutant hermaphrodite expressing mitoGFP in body wall muscle. mitoGFP labels muscle mitochondrial tubules. (B) Image of a single body wall muscle in a transgenic *vpr-1(tm1441)* mutant hermaphrodite expressing mitoGFP. (C) Close-up image of the boxed region from panel B showing horizontal and vertical cross-sections. Arrowheads indicate lipid-like droplets. Asterisks indicate nucleus. Bar in A, 1 µm; Bars in B and C, 10 µm.
(TIF)

Figure S2 Transmission electron micrographs of wild-type and mutant adults. Micrographs are transverse sections showing the cuticle (C) and body wall muscle sarcomeres (S) with hypodermis sandwiched in between. A lipid droplet within the hypodermis (HL) is seen in the wild-type panel. The intestine is filled with electron dense and opaque lipid droplets (IL). Yolk lipoprotein complexes (Y) are found between muscle and intestinal tissues (also see Figure S4). Notice that yolk is electron dense, whereas muscle lipid droplets (ML) are opaque. N, muscle nucleus. Bars, 1 µm.
(TIF)

Figure S3 Sudan Black B staining in wild type and *vpr-1* mutants. 1-day-old adult wild-type and *vpr-1(tm1411)* hermaphrodites were stained using Sudan Black B. Arrowheads indicate fat droplets in body wall muscle. Anterior is to the left in all panels. Boxed regions are magnified 5× below. Low magnification bars, 50 µm; high magnification bars, 10 µm.
(TIF)

Figure S4 Yolk lipoprotein complex distribution in wild-type and *vpr-1* mutants. (A) Yolk distribution visualized with the *vit-2p::vit-2::gfp* transgene. *vpr-1* mutants accumulate yolk in the pseudocoelom due to failure of oocyte differentiation. GFP uptake is not observed in peripheral tissues. (B) Close-up image showing muscle fat droplets (arrows) in *vpr-1* mutants. Bar, 5 µm.
(TIF)

Figure S5 ER stress assays in wild-type and *vpr-1* mutant worms. (A) Integrated transgenic lines expressing GFP under the *hsp-4* promoter *(hsp-4p::GFP)* with and without tunicamycin treatment, which induces ER stress. Anterior is to the left in all panels. Bar, 5 mm. (B) Tunicamycin sensitivity in wild-type and *vpr-1(tm1411)* mutants hermaphrodites. Y-axis indicates the percentage of worms that developed to the adult stage in the presence of 5 µg/ml tunicamycin. Error bars represent SD. Three independent measurements were performed.
(TIF)

Figure S6 Effect of sperm presence on muscle fat droplets and mitochondria. (A) DIC and fluorescent images of muscle in live 3-day-old *vpr-1* mutant hermaphrodite worms fed Bodipy-FAs. Mating with wild type (WT) males provides sperm into the uterus. Sperm presence did not affect the sterility or muscle mitochondrial morphology of *vpr-1* mutants (data not shown). Anterior is to the left in all panels. Arrowheads indicate lipid-like droplets. Bar, 5 µm. (B) DIC and fluorescent images of muscle in live transgenic *fog-3(q443)/hT2* hermaphrodites containing sperm and unmated *fog-3(q443)* mutants without sperm. Muscle mitochondrial tubules were visualized using mitoGFP. Arrowheads indicate lipid-like droplets. Asterisks indicate nucleus. Bar, 5 µm.
(TIF)

Figure S7 Effect of ARX-2/Arp2 overexpression on muscle fat droplets and mitochondria. DIC and fluorescent images of muscle in live transgenic wild-type hermaphrodites expressing *arx-2* under control of the muscle specific *myo-3* promoter. Muscle mitochondrial tubules were visualized using mitoGFP. Notice that muscle mitochondrial morphology closely resembles the morphology seen in *vpr-1* mutants [15]. See Figures 7A, S6B and [15] for controls. Arrows indicate lipid-like droplets. Asterisks indicate nucleus. Bar, 5 µm.
(TIF)

Table S1 Primers used for RT-qPCR in mice.
(TIF)
