# Supplementary figures and images for: VAPB/ALS8 MSP Ligands Regulate Striated Muscle Energy Metabolism Critical for Adult Survival in Caenorhabditis elegans
Source: PLoS Genet. 2013 Sep 5;9(9):e1003738. doi: 10.1371/journal.pgen.1003738 (PMC3764199; doi:10.1371/journal.pgen.1003738)

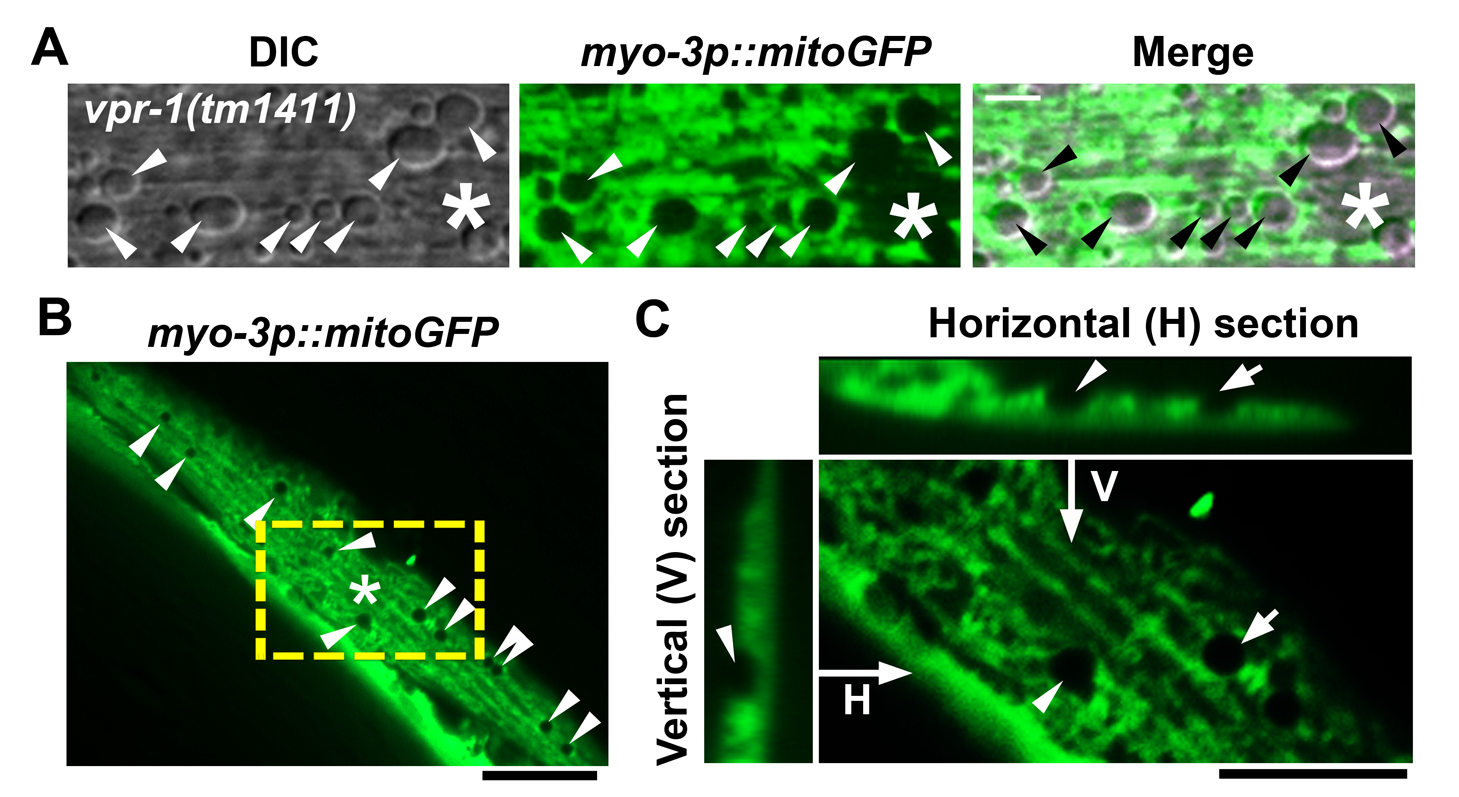

Supplement: Figure S1 — Lipid-like droplets and mitochondria in vpr-1 mutant striated muscle. (A) Close-up image of a live transgenic vpr-1(tm1441) mutant hermaphrodite expressing mitoGFP in body wall muscle. mitoGFP labels muscle mitochondrial tubules. (B) Image of a single body wall muscle in a transgenic vpr-1(tm1441) mutant hermaphrodite expressing mitoGFP. (C) Close-up image of the boxed region from panel B showing horizontal and vertical cross-sections. Arrowheads indicate lipid-like droplets. Asterisks indicate nucleus. Bar in A, 1 µm; Bars in B and C, 10 µm. (TIF) [file pgen.1003738.s001.tif]

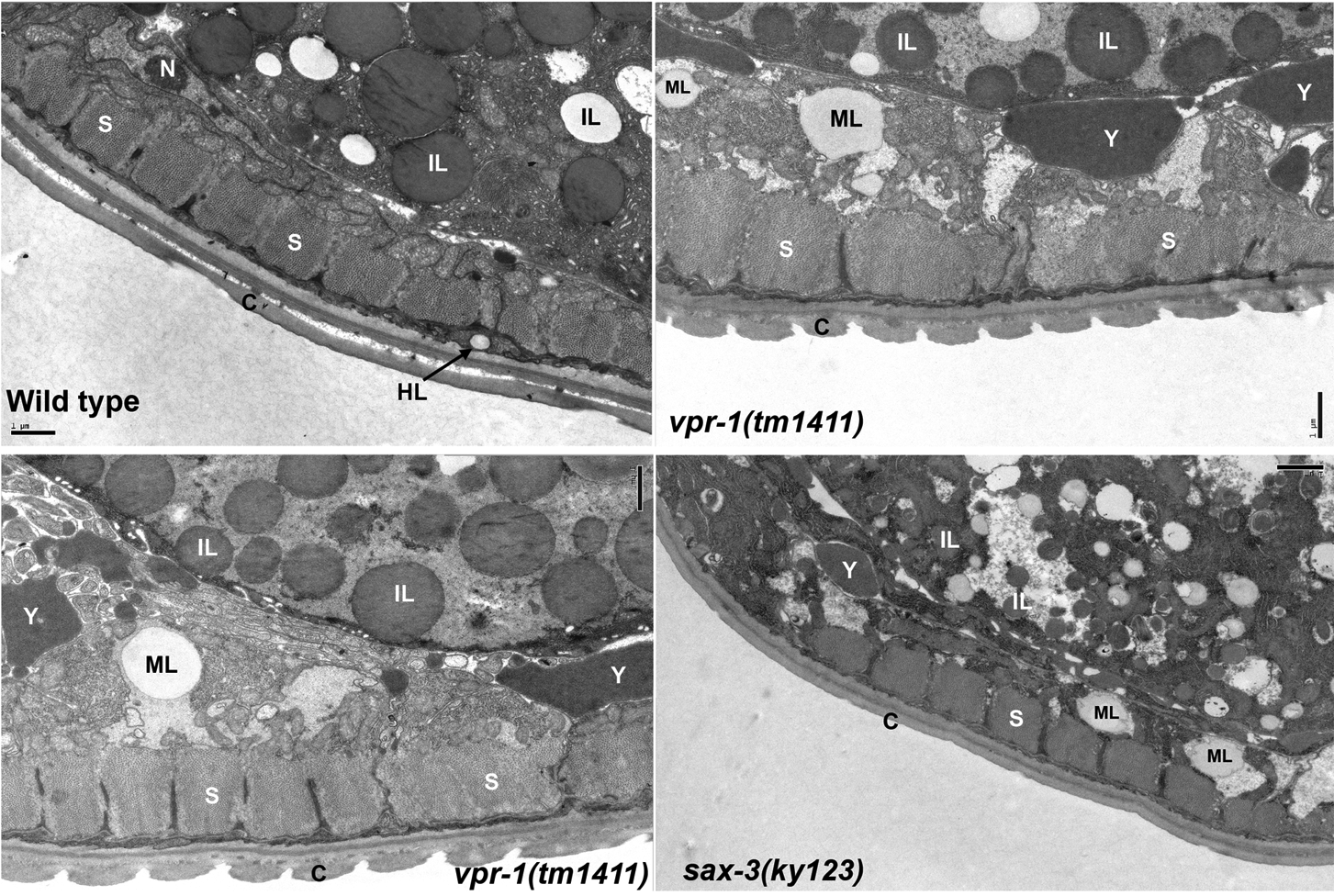

Supplement: Figure S2 — Transmission electron micrographs of wild-type and mutant adults. Micrographs are transverse sections showing the cuticle (C) and body wall muscle sarcomeres (S) with hypodermis sandwiched in between. A lipid droplet within the hypodermis (HL) is seen in the wild-type panel. The intestine is filled with electron dense and opaque lipid droplets (IL). Yolk lipoprotein complexes (Y) are found between muscle and intestinal tissues (also see Figure S4). Notice that yolk is electron dense, whereas muscle lipid droplets (ML) are opaque. N, muscle nucleus. Bars, 1 µm. (TIF) [file pgen.1003738.s002.tif]

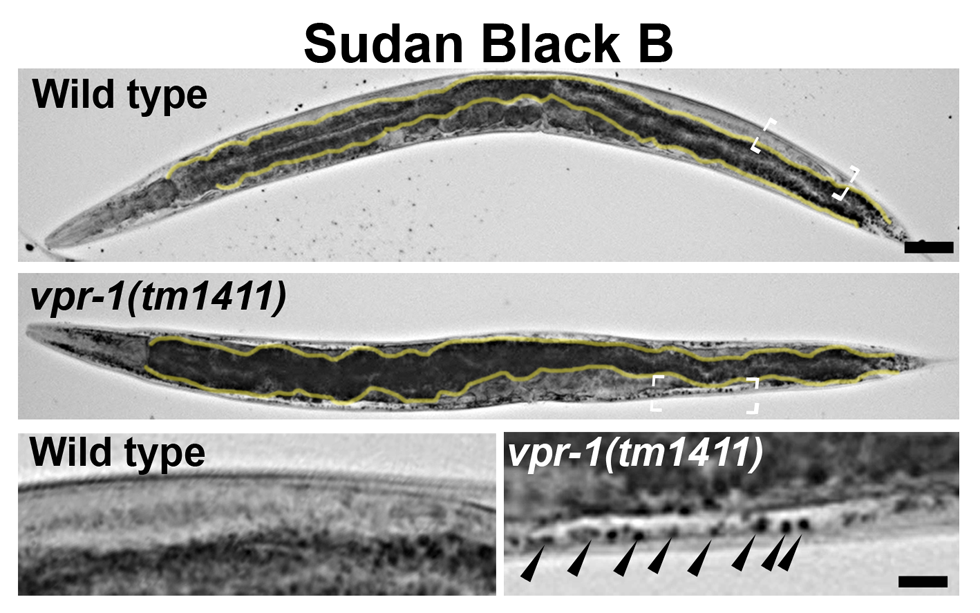

Supplement: Figure S3 — Sudan Black B staining in wild type and vpr-1 mutants. 1-day-old adult wild-type and vpr-1(tm1411) hermaphrodites were stained using Sudan Black B. Arrowheads indicate fat droplets in body wall muscle. Anterior is to the left in all panels. Boxed regions are magnified 5× below. Low magnification bars, 50 µm; high magnification bars, 10 µm. (TIF) [file pgen.1003738.s003.tif]

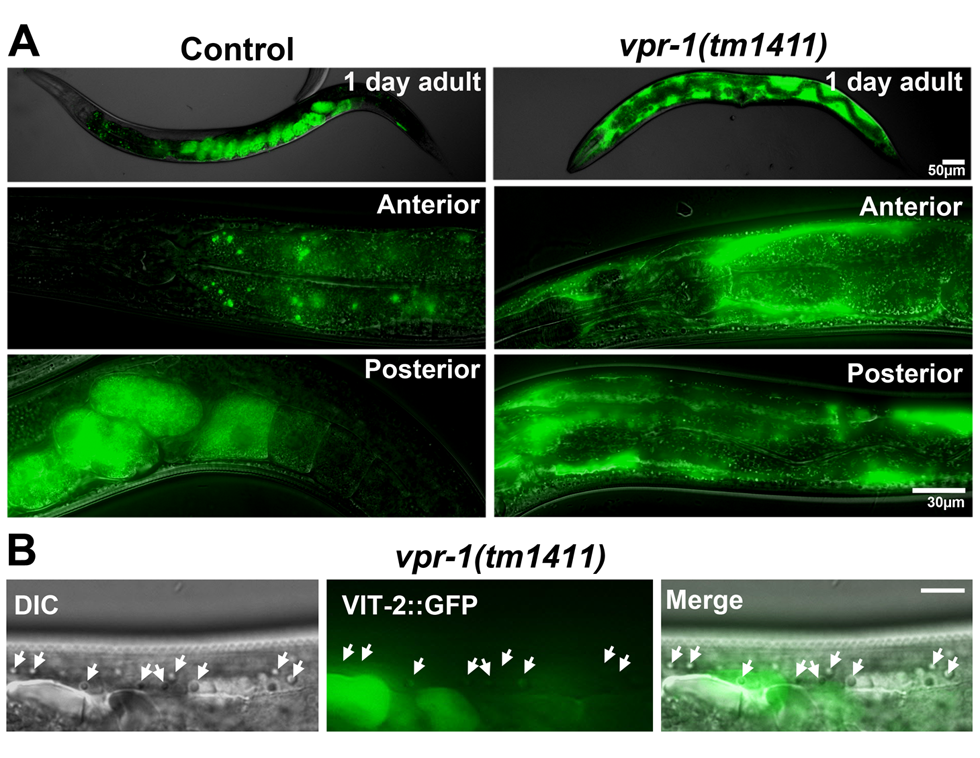

Supplement: Figure S4 — Yolk lipoprotein complex distribution in wild-type and vpr-1 mutants. (A) Yolk distribution visualized with the vit-2p::vit-2::gfp transgene. vpr-1 mutants accumulate yolk in the pseudocoelom due to failure of oocyte differentiation. GFP uptake is not observed in peripheral tissues. (B) Close-up image showing muscle fat droplets (arrows) in vpr-1 mutants. Bar, 5 µm. (TIF) [file pgen.1003738.s004.tif]

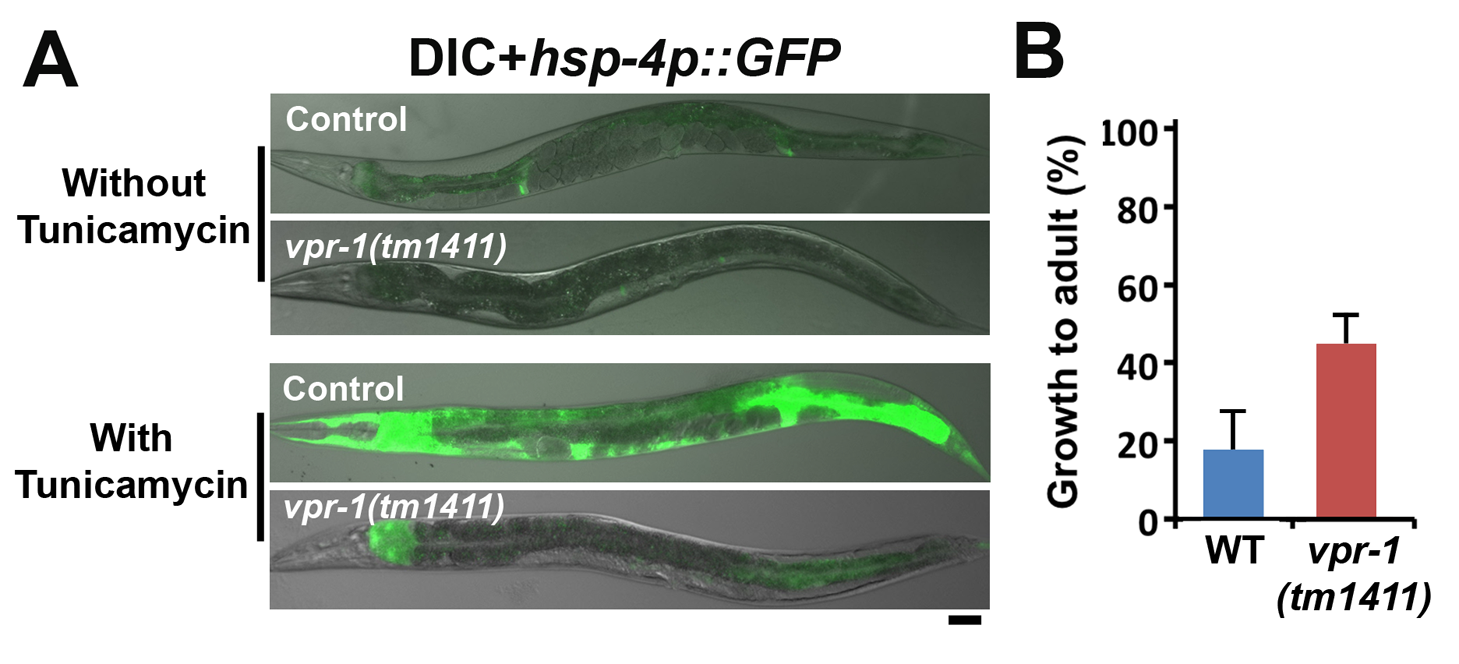

Supplement: Figure S5 — ER stress assays in wild-type and vpr-1 mutant worms. (A) Integrated transgenic lines expressing GFP under the hsp-4 promoter (hsp-4p::GFP) with and without tunicamycin treatment, which induces ER stress. Anterior is to the left in all panels. Bar, 5 mm. (B) Tunicamycin sensitivity in wild-type and vpr-1(tm1411) mutants hermaphrodites. Y-axis indicates the percentage of worms that developed to the adult stage in the presence of 5 µg/ml tunicamycin. Error bars represent SD. Three independent measurements were performed. (TIF) [file pgen.1003738.s005.tif]

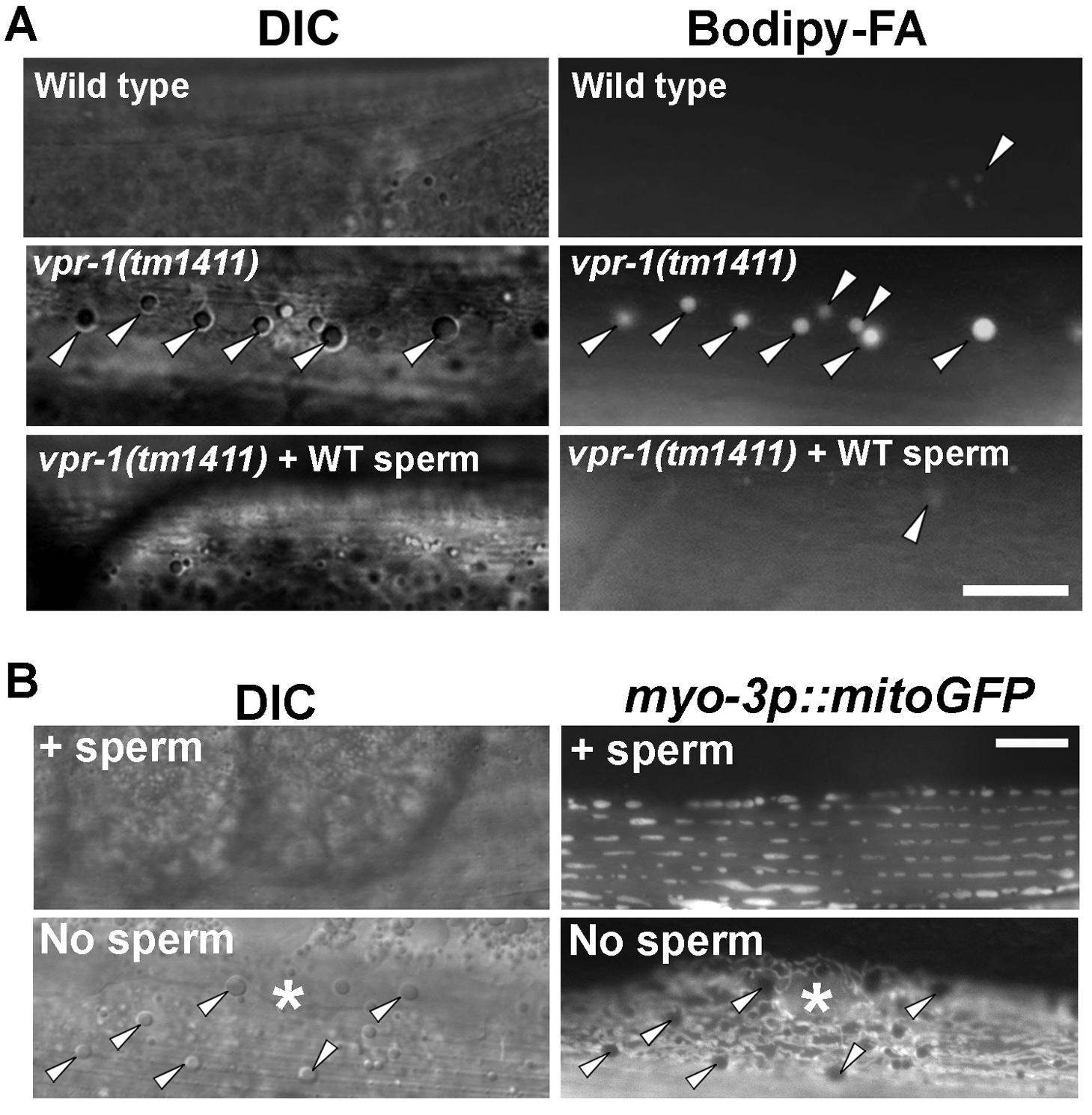

Supplement: Figure S6 — Effect of sperm presence on muscle fat droplets and mitochondria. (A) DIC and fluorescent images of muscle in live 3-day-old vpr-1 mutant hermaphrodite worms fed Bodipy-FAs. Mating with wild type (WT) males provides sperm into the uterus. Sperm presence did not affect the sterility or muscle mitochondrial morphology of vpr-1 mutants (data not shown). Anterior is to the left in all panels. Arrowheads indicate lipid-like droplets. Bar, 5 µm. (B) DIC and fluorescent images of muscle in live transgenic fog-3(q443)/hT2 hermaphrodites containing sperm and unmated fog-3(q443) mutants without sperm. Muscle mitochondrial tubules were visualized using mitoGFP. Arrowheads indicate lipid-like droplets. Asterisks indicate nucleus. Bar, 5 µm. (TIF) [file pgen.1003738.s006.tif]

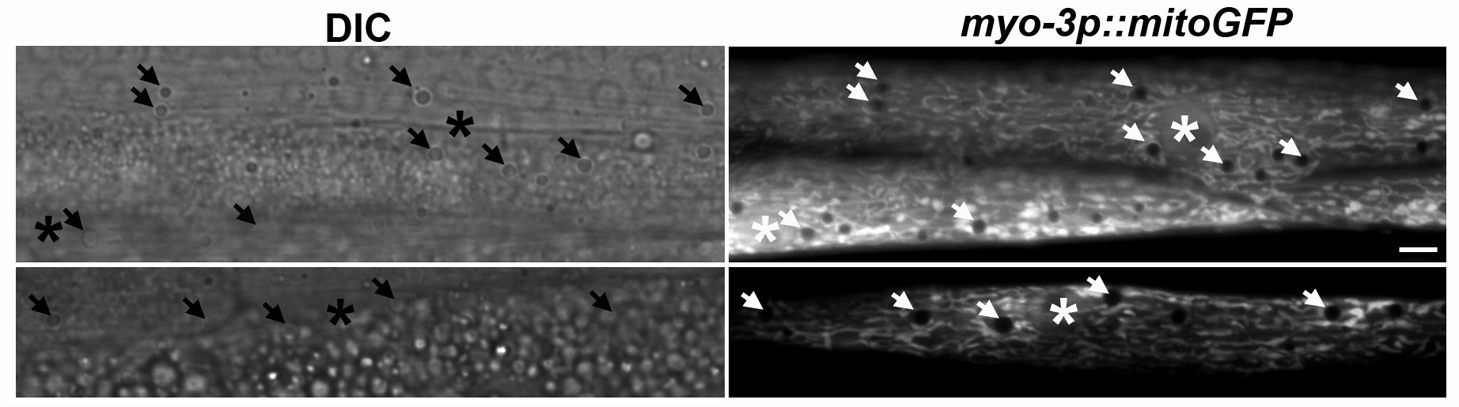

Supplement: Figure S7 — Effect of ARX-2/Arp2 overexpression on muscle fat droplets and mitochondria. DIC and fluorescent images of muscle in live transgenic wild-type hermaphrodites expressing arx-2 under control of the muscle specific myo-3 promoter. Muscle mitochondrial tubules were visualized using mitoGFP. Notice that muscle mitochondrial morphology closely resembles the morphology seen in vpr-1 mutants [15]. See Figures 7A, S6B and [15] for controls. Arrows indicate lipid-like droplets. Asterisks indicate nucleus. Bar, 5 µm. (TIF) [file pgen.1003738.s007.tif]

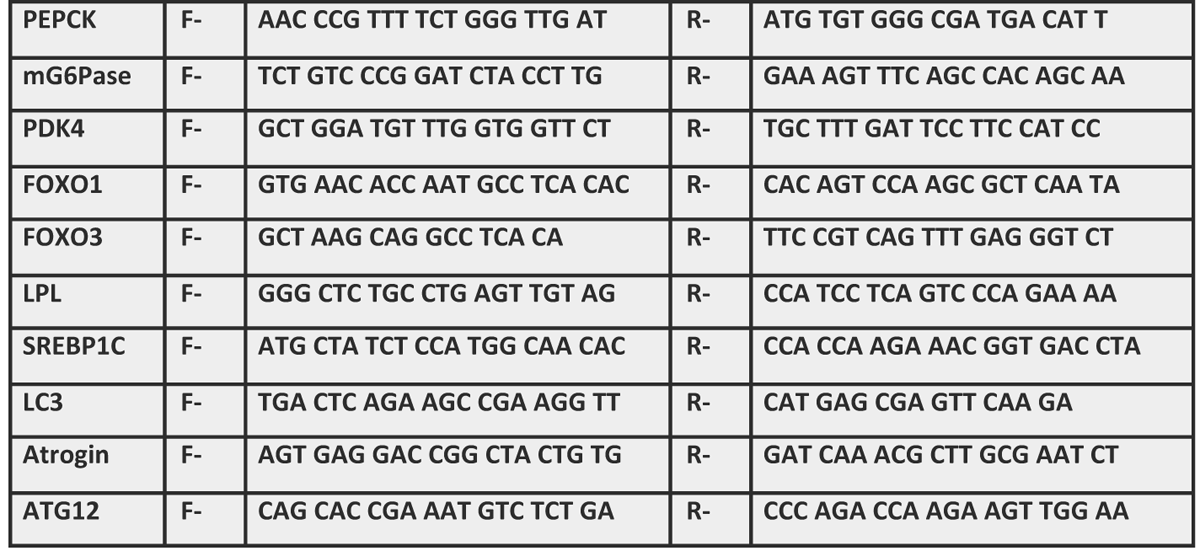

Supplement: Table S1 — Primers used for RT-qPCR in mice. (TIF) [file pgen.1003738.s008.tif]
